# Noninvasive ultrasound stimulation to treat myocarditis through splenic neuro-immune regulation

**DOI:** 10.1186/s12974-023-02773-2

**Published:** 2023-04-17

**Authors:** Tianshu Liu, Yanan Fu, Jiawei Shi, Shukun He, Dandan Chen, Wenqu Li, Yihan Chen, Li Zhang, Qing Lv, Yali Yang, Qiaofeng Jin, Jing Wang, Mingxing Xie

**Affiliations:** 1 Department of Ultrasound Medicine, Union Hospital, Tongji Medical College, Huazhong University of Science and Technology, Wuhan, 430022 China; 2 Hubei Province Clinical Research Center for Medical Imaging, Wuhan, 430022 China; 3 Hubei Province Key Laboratory of Molecular Imaging, Wuhan, 430022 China

**Keywords:** Low-intensity pulsed ultrasound, Experimental autoimmune myocarditis, Cholinergic anti-inflammatory pathway, CCR2+ macrophage, CD4+ Treg cell

## Abstract

**Background:**

The cholinergic anti-inflammatory pathway (CAP) has been widely studied to modulate the immune response. Current stimulating strategies are invasive or imprecise. Noninvasive low-intensity pulsed ultrasound (LIPUS) has become increasingly appreciated for targeted neuronal modulation. However, its mechanisms and physiological role on myocarditis remain poorly defined.

**Methods:**

The mouse model of experimental autoimmune myocarditis was established. Low-intensity pulsed ultrasound was targeted at the spleen to stimulate the spleen nerve. Under different ultrasound parameters, histological tests and molecular biology were performed to observe inflammatory lesions and changes in immune cell subsets in the spleen and heart. In addition, we evaluated the dependence of the spleen nerve and cholinergic anti-inflammatory pathway of low-intensity pulsed ultrasound in treating autoimmune myocarditis in mice through different control groups.

**Results:**

The echocardiography and flow cytometry of splenic or heart infiltrating immune cells revealed that splenic ultrasound could alleviate the immune response, regulate the proportion and function of CD4+ Treg and macrophages by activating cholinergic anti-inflammatory pathway, and finally reduce heart inflammatory injury and improve cardiac remodeling, which is as effective as an acetylcholine receptor agonists GTS-21. Transcriptome sequencing showed significant differential expressed genes due to ultrasound modulation.

**Conclusions:**

It is worth noting that the ultrasound therapeutic efficacy depends greatly on acoustic pressure and exposure duration, and the effective targeting organ was the spleen but not the heart. This study provides novel insight into the therapeutic potentials of LIPUS, which are essential for its future application.

**Graphical Abstract:**

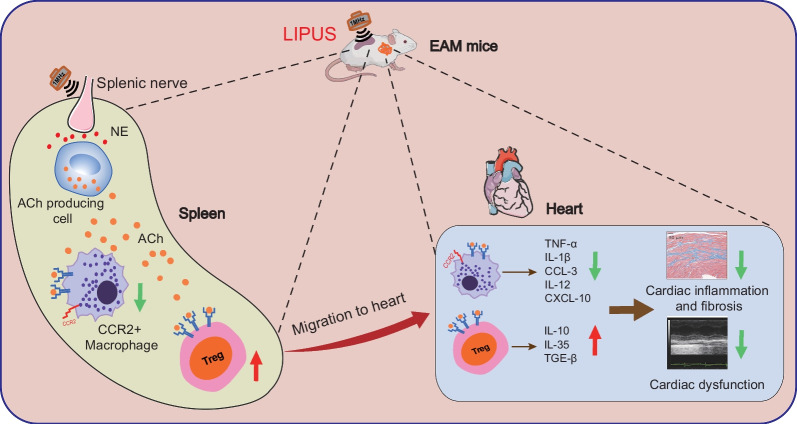

**Supplementary Information:**

The online version contains supplementary material available at 10.1186/s12974-023-02773-2.

## Background

Myocarditis is an inflammatory heart disease induced by many different causes and may result in myocardial injury, dilated cardiomyopathy and chronic heart failure [1]. The complex and aberrant autoimmune mechanisms play an indispensable role in its pathological process. Despite its high mortality and severe harm, only symptomatic treatment is currently available in treating myocarditis.

Cholinergic anti-inflammatory pathway (CAP), a reflex arc that induces vagus nerve stimulation (VNS) signaling that originates in the brain and terminates in the spleen, has been widely studied for modulating the immune response. CAP has been shown to benefit animals in models of ischemia–reperfusion injury, hepatic injury, sepsis, endotoxemia, and the response of humans injected with lipopolysaccharide, by reducing immune cell activation and inflammatory cytokine production [2–5]. Provided suitable stimulus tools, CAP would be a promising strategy for many other inflammatory diseases, including myocarditis.

Currently, CAP can be stimulated pharmacologically with nicotinic or α7-nAChR agonists (i.e., GTS-21) or non-pharmacological electrical stimulation. Because the action of nicotine agonists produces widespread acetylcholine (ACh), it produces lots of side effects when administered systemically and usually has a narrow therapeutic window [6]. As a result, more studies have turned to non-pharmacological options such as implantable vagus nerve electrode cuffs, making the stimulation more specific. However, it is difficult to stimulate the CAP precisely in the spleen since the implanted electrodes are limited to stimulating the large nerves, leading to the activation of other organs and inducing side effects on multiple physiological and life-sustaining functions [7]. Most importantly, implanting electrodes into the spleen for VNS will be invasive and challenging clinically and technically because of the anatomical structure of the splenic nerve, which enters the spleen as a dense vascular plexus along with the splenic artery and continues to course along arterial and venous branches under the capsule. Therefore, developing advanced VNS tools that stimulate the CAP non-invasively and accurately would promote the pathway into clinical implementation to treat many diseases.

In the past decades, ultrasound has proved effective and safe in BBB (blood–brain barrier)-opening, targeted drug/gene delivery with microbubbles or other ultrasound-responsive carriers [8–12]. Ultrasound has become increasingly appreciated for neuronal modulation non-invasively [13]. Focused ultrasound has been reported to both excite and inhibit neural activity reversibly [14]. Evidence suggests the potential protective role of therapeutic low-intensity pulsed ultrasound (LIPUS) in acute and chronic inflammation via modulation of CAP, such as arthritis and lipopolysaccharide (LPS)-induced sepsis [15, 16]. However, the biological mechanism underlying the treatment is not clear enough. Adaptive and cellular immunotherapy is essential in autoimmune myocarditis treatment [17]. Herein, we proposed that ultrasound VNS may be attributed to the therapy of myocarditis by modulating the immune response via CAP and relieving symptoms and inhibiting the inflammatory process, and attempted to elucidate the underlying immune mechanisms involved in the beneficial effects of the LIPUS.

## Methods

### EAM induction and LIPUS stimulation

**Fig. 1:**
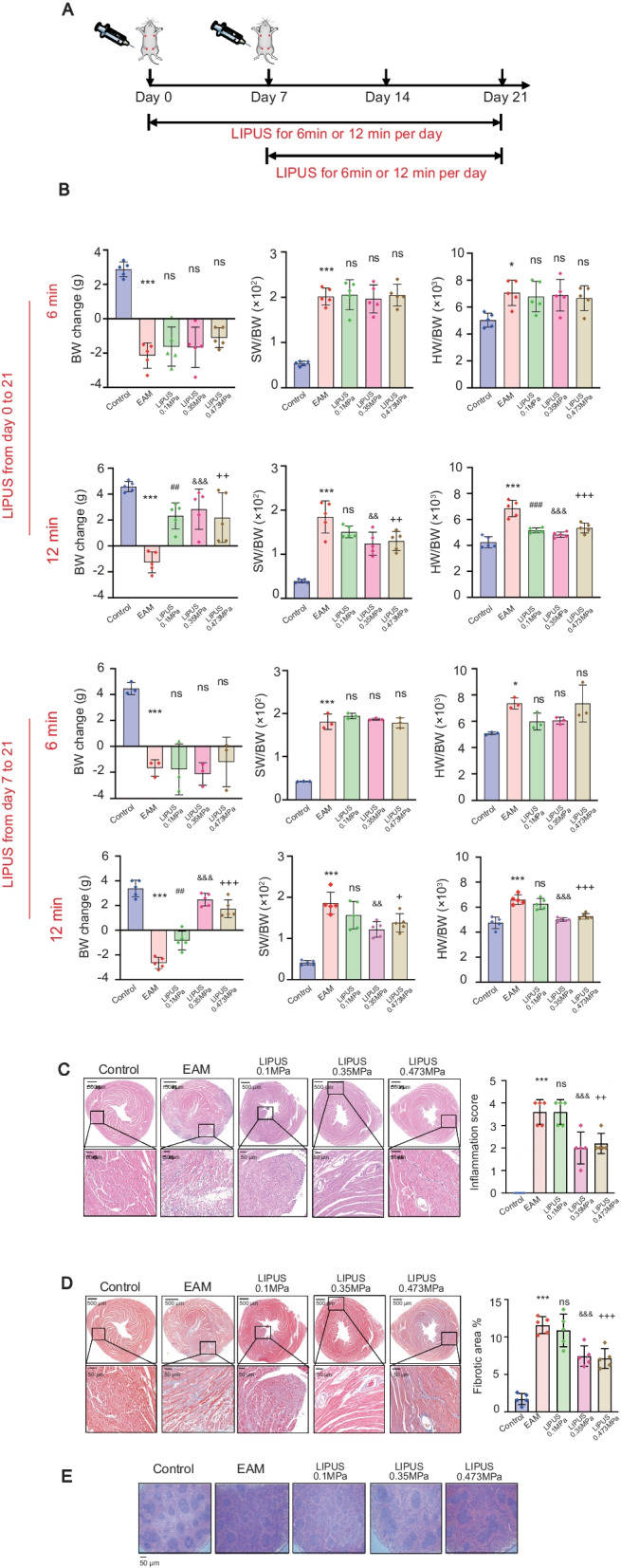
The therapeutic effect of LIPUS on myocarditis and optimal parameter screening. **A** Timeline of an EAM intervention experiment performed in the presented study in which animals were immunized with 200 μg of cardiac-specific peptide on day 0 and 7, then treated with focused ultrasound that targeted the spleen on day 0 or day 7. **B** Data from different experimental conditions (1.0 MHz US at 0.1 MPa, 0.35 MPa, 0.473 MPa; 1 s on /5 s off burst for 6 or 12 min per day) were shown. On the final day of the experiment, BW change, SW/BW and HW/BW of ultrasound treated animals in different experimental periods day 0–21 or day 7–21. **C** HE staining for the degree of inflammatory cell infiltration of EAM hearts. Scale: 500 μm (top) and 50 μm (bottom). (D) MASSON staining determined collagen deposition in the inflammatory region. Scale: 500 μm (top) and 50 μm (bottom). Representative HE and MASSON staining images of indicated groups. Bar graphs on the right in C and D show quantitation of data (*n* = 5). **E** HE staining of spleen tissue sections. *n* = 5. Scale: 50 μm. ^*^ Represents control vs. EAM; ^#^ represents LIPUS 0.1 MPa vs. EAM; ^&^ represents LIPUS 0.35 MPa vs. EAM; ^+^ represents LIPUS 0.473 MPa vs. EAM. (Data were shown as the mean ± SD, ordinary one-way analysis of variance, Tukey's multiple comparisons test; ^*^P or ^+^*P* < 0.05, ^##^*P*, ^&&^*P* or ^++^*P* < 0.01, ^***^P, ^###^*P*, ^&&&^*P* or ^+++^*P* < 0.001). BW: body weight; SW/BW: spleen weight to body weight; HW/BW: heart weight to body weight; EAM: experimental autoimmune myocarditis; HE: hematoxylin and eosin

The mouse model of experimental autoimmune myocarditis (EAM) was prepared to mimic human inflammatory heart disease [18, 19]. Male BALB/c mice aged 6–7 weeks (weighing 18–20 g) were purchased from HFK Bioscience CO., LTD. (Beijing, China). Mice were immunized with cardiac-specific peptide (MHC-α_614–629_: Acetyl-SLKLMATLFSTYAS) purchased from GL Biochem (Shanghai) Ltd. Briefly, the peptide was first dissolved in saline and emulsified with complete Freund’s adjuvant (CFA) in a 1:1 ratio. Then, 200 μg peptide in 0.2 ml of the emulsion was injected subcutaneously into one side of the axillary and inguinal lymph node region on day 0, and the same dosing was injected subcutaneously into the opposite side on day 7. For LIPUS stimulation, mice were anesthetized with isoflurane before LIPUS stimulation. Fur from the left armpit to the abdomen and back of the mice was shaved and removed using a depilatory. Then, the mice were placed on the operating table in the correct decubitus position. The spleen was visible through the thin skin. The targeted LIPUS on spleen was about halfway between the shoulder and hip joints of mice (Additional file 2). The prewarmed gel was then placed on the depilated skin for LIPUS stimulation. There is a semi-liquid acoustic coupling gel between the transducer and the skin of the mice to help the ultrasound energy being delivered into the spleen. Therefore, the transducer would have not cause direct pressure on the mice during stimulation (Additional file 4). The system consists of a Function Generator (Tektronix AFG3051C), a power amplifier (AG 1020, T&C Power Conversion, Inc.) and a concave transducer (OLYMPUS V302) with a custom-designed focusing cone filled with agarose gel (1.5%, w/v). The LIPUS parameters applied as follows: 1 MHz at 0.1 MPa (LIPUS 0.1 MPa), 0.35 MKPa (LIPUS 0.35 MPa), 0.473 MPa (LIPUS 0.473 MPa), 1 s on/5 s off bursts for 6 min or 12 min stimulation during days 0 to 21 or days 7 to 21 (Table 1 and Fig. 1A). The chronic treatment experiment was carried out from days 7 to 56. In addition, the splenic nerve resection was performed as follows: forceps were used to blunt-isolate the spleen away from the peritoneal cavity so that the vasculature trees were clearly exposed (Fig. 5B-a). Under a dissection microscope, the location of the splenic nerves (black arrow) accompanying the vasculature trees (white arrow) was identified (Fig. 5B-b). Absolute ethanol was repeatedly applied with cotton tips to those vasculature trees in order to deplete the splenic nerve fibers that run along them (Fig. 5B-c). The abdominal wall and incision were then closed with sutures. Mice were allowed to recover 6 days prior to the LIPUS treatment (Fig. 5B-d).

**Table 1:** Ultrasound neuromodulation parameters

Model	Burst
Probe	Olympus v302 1.0 MHz/1″F = 2.0″PTF 1,169,880
Frequency (MHz)	1.0
Pressure (kPa)	100 kPa; 350 kPa; 473 kPa
Voltage (mV)	94.7 mV; 409.3 mV; 1000 mV
Pulse duration (s)	1
Pulse repetition period (s)	6
Duty cycle	16.7%
Stimulation time	12 min or 6 min

The system consists of a single element focused ultrasound transducer, a power amplifier and a function generator. The function generator produces a pulsed sinusoidal waveform. The pulse center frequency was 1.0 MHz; the pulse repetition period was 6 s; the pulse duration was 1 s. The duty cycle is the fraction of time that the ultrasound stimulus is active within each pulse repetition period, which was 16.7% in our study. The total stimulation time was 12 min or 6 min. The different ultrasound pressures were set as 0.1 MPa, 0.35 MPa, 0.473 MPa. The corresponding voltage was 94.7 mV, 409.3 mV and 1000 mV, respectively

### Measurement of echocardiography and blood pressure

Transthoracic echocardiography of mice was performed on day 21 or 56 using the high-resolution ultrasound imaging system equipped with a 30-MHz transducer (Vevo 3100, Fujifilm Visual Sonics, Toronto, Canada). The Softron BP-2010 Series, a noninvasive tail-cuff system, was utilized to measure systolic blood pressure (BP), diastolic BP and mean BP. All mice were first trained to stay quietly in a restrainer placed on a warm pad for 15 min before the measurement.

### Histological examination

To assess myocardial inflammation and fibrosis, the heart sections were cut into cross sections and stained with hematoxylin and eosin (H&E) and MASSON. The inflammation score was as follows: grade 0, no inflammatory infiltrates; grade 1, < 25% of a cross section involved; grade 2, 25–50% of a cross section involved; grade 3, 50–75% of a cross section involved; > 75% of a cross section involved [20]. To determine the spatial location of nerve endings in the spleen, we measured the distribution pattern of the presynaptic vesicle-associated protein synaptophysin (#36406, CST, USA) and ChAT (#12420, ABclonal, China), α7nAChR (#1588, ABclonal, China).

### Quantitative real-time PCR analysis

Total messenger RNA was extracted using Trizol ®reagent (Invitrogen, USA) and converted into cDNA using PrimeScript RT Reagent Kit (Takara Biotechnology, Japan). Real-time PCR analyses were performed on a Bio-rad CXF CONNECT Detector system using SYBR green master mix (Takara Biotechnology, Japan). β-actin was used as the internal reference. Details are in Additional file 8: Table S3.

### Cardiac-infiltrating cell isolation

Cardiac-infiltrating cells were isolated from mice hearts using minor modifications of the method described previously [18]. In brief, hearts were collected and perfused with HEPES buffer (20 mM HEPES, 130 mM NaCl, 3 mM KCl, 1 mM NaH2PO4, 4.5 mM glucose, pH = 7.4) and then minced and digested in 0.1% collagenase B solution (C6885, Sigma, USA; 1 mg/ml in HEPES buffer) at 37 °C. The digested cell suspension was then filtered through 40-μm cell strainers to prepare a single-cell suspension. The cardiac lymphocytes isolated by density gradient centrifugation were cultured in a complete RPMI-1640 medium with 10% fetal bovine serum. The number of immune cells extracted is much less than that from the spleen. Therefore, according to the actual number of cells isolated, we will combine the hearts of two or three mice to ensure that there is a sufficient number of similar suspended single cells for flow cytometry detection each time, which is conducive to subsequent statistical analysis and guarantee the quality of experimental results.

### Flow cytometry analysis

The resulting single-cell suspension isolated from the cardiac and spleen was immune-stained with various combinations of fluorescing antibodies. Cardiac and splenic immune cell subsets gating strategy is shown in additional file 3. Details are in Additional file 8: Table S4.

### ELISA

For the enzyme-linked immunosorbent assay (ELISA), spleen samples were collected. According to the manufacturer's instructions, the ACh and norepinephrine (NE) levels were quantified using ELISA kits (Boster Biological Technology, China).

### Cell sorting and RNA sequencing

12 EAM mice with or without LIPUS stimulation (1 MHz at 350 kPa using 1 s on/5 s off bursts for 12 min per day from days 7 to 21) were prepared for transcriptome sequencing experiments. Spleens were processed into single-cell suspensions. CD4+ T cell or CD11b+ macrophage was selected using an isolation kit (#480005 and #480109, Biolegend, USA). Total RNA was extracted using TRIzol Reagent according to the manufacturer’s instructions (Invitrogen, USA), and genomic DNA was removed using DNase I (Takara, Japan). Then RNA quality was determined by 2100 Bioanalyser (Agilent, USA) and quantified using the ND-2000 (NanoDrop Technologies). RNA-seq transcriptome library of CD11b+ macrophage was prepared following TruSeq™ RNA sample preparation Kit from Illumina (San Diego, CA) using 1 μg of total RNA. Paired-end RNA-seq sequencing library was sequenced with the Illumina HiSeq xten/NovaSeq 6000 sequencer. RNA-seq transcriptome library of CD4+ T cell was prepared following Clontech-SMART-SeqTM v4 UltraTM Low Input RNA Kit for Sequencing using 10 ng of total RNA. Then the synthesized cDNA was conducted into library according to Illumina’s library construction protocol. Finally, the paired-end RNA-seq sequencing library was sequenced with the Illumina NovaSeq 6000 sequencer. To identify differential expression genes (DEGs) between different treatments, we calculated the raw counts of each transcript according to the RSEM (RNA-Seq by Expectation–Maximization) method. Normalized gene expression was computed using Transcripts per million (TPM) reads to eliminate the effects of gene length and sequencing depth. Essentially, differential expression analysis was performed using the DESeq223, and Genes with |log2FC|> 0.58 and False Discovery Rate (FDR) ≤ 0.05 were considered significant DEGs. In addition, Gene Set Enrichment Analysis (GSEA) of Kyoto Encyclopedia of Genes and Genomes (KEGG) was performed to identify which pathways were significantly enriched at FDR ≤ 0.05 compared with the whole-transcriptome background.

### Statistical analysis

Statistical analysis was performed using GraphPad Prism 8.0 (GraphPad Software Inc., California, USA). We performed Student’s t-test for two-group comparison and one-way analysis of variance (ANOVA), followed by Tukey’s multiple comparison test for comparison of multiple groups. All data are presented as the mean value ± standard deviation (mean ± SD). In all tests, differences with *P* < 0.05 were considered statistically significant.

## Results

### The therapeutic effect of LIPUS on myocarditis and optimal parameter screening

Generally, the body weight (BW) of EAM mice reduced gradually, representing an increased systemic inflammatory response. The ratio of spleen weight to body weight (SW/BW) in untreated EAM mice was increased as expected. An increase in heart weight to body weight ratio (HW/BW) indicates increased myocarditis severity. The above characteristic indexes were used to monitor myocarditis progress and optimize the ultrasound parameters (Fig. 1).

To explore the therapeutic effect of LIPUS, mice were anesthetized and placed on the operating table in the correct decubitus position. To investigate the therapeutic efficacy of various parameters, daily splenic ultrasound treatment was performed with a 1-MHz focused transducer for 6 or 12 min over a 15-day or 21-day period at 0.1, 0.35, 0.473 MPa, respectively. 1 s on/5 s off duty cycle was used in all experiments (Table 1). As shown in Fig. 1A and B, ultrasound stimulation prior to the successful establishment of EAM models from day 0 to 21 did not advance the time point of weight reversal in mice. Enough acoustic pressure and stimulation duration were demonstrated necessary for EAM treatment. In the same treatment period (day 0–21 or 7–21), the therapeutic efficacy of 12 min stimulation was better than that of 6 min stimulation, which showed increased BW and reduced SW/BW and HW/BW ratios (Fig. 1B). Eventually, it revealed little difference between ultrasound stimulation starting on day 0 and day 7, in the stimulation duration of 6 or 12 min. Moreover, since the model of EAM has not been built successfully on day 0, prophylactic treatment of myocarditis is not clinically feasible. A more reasonable situation is to start the ultrasound treatment on day 7. Hence, ultrasound stimulation for 12 min from day 7 to 21 was considered an optimal parameter in the following study.

### LIPUS stimulation targeting spleen ameliorates cardiac inflammation and injury induced by EAM

At the histological levels, the inflammation score (HE staining in Fig. 1C) and pathological cardiac fibrosis (MASSON staining in Fig. 1D) of hearts in LIPUS groups were significantly attenuated, specifically in LIPUS-0.35 MPa group. Ultrasound wave may produce certain cavitation efficiency and thermal efficiency [21]. Spleen HE staining is to observe the safety of focused ultrasound stimulation on target organs, which shows whether the spleen has pathological damage such as hemorrhage and cell necrosis. The results in Fig. 1E showed that there was no significant difference in the microstructure of the spleen among the groups, that is, the ultrasound parameters used in this study were safe enough. In the above results, LIPUS stimulation targeting the spleen ameliorated cardiac inflammation and injury induced by EAM. Treatment response depended on acoustic pressure, and immunohistochemistry showed the effective pressure parameters were 0.35 MPa or higher. Following the principle of as low as reasonably achievable in the medical ultrasound, we have successfully identified an effective treatment parameter setting using the 1 MHz frequency at 0.35 MPa with a 1 s on /5 s off duty cycle by targeting the spleen.

### LIPUS therapy improves cardiac function and modifies the immune response of EAM

**Fig. 2:**
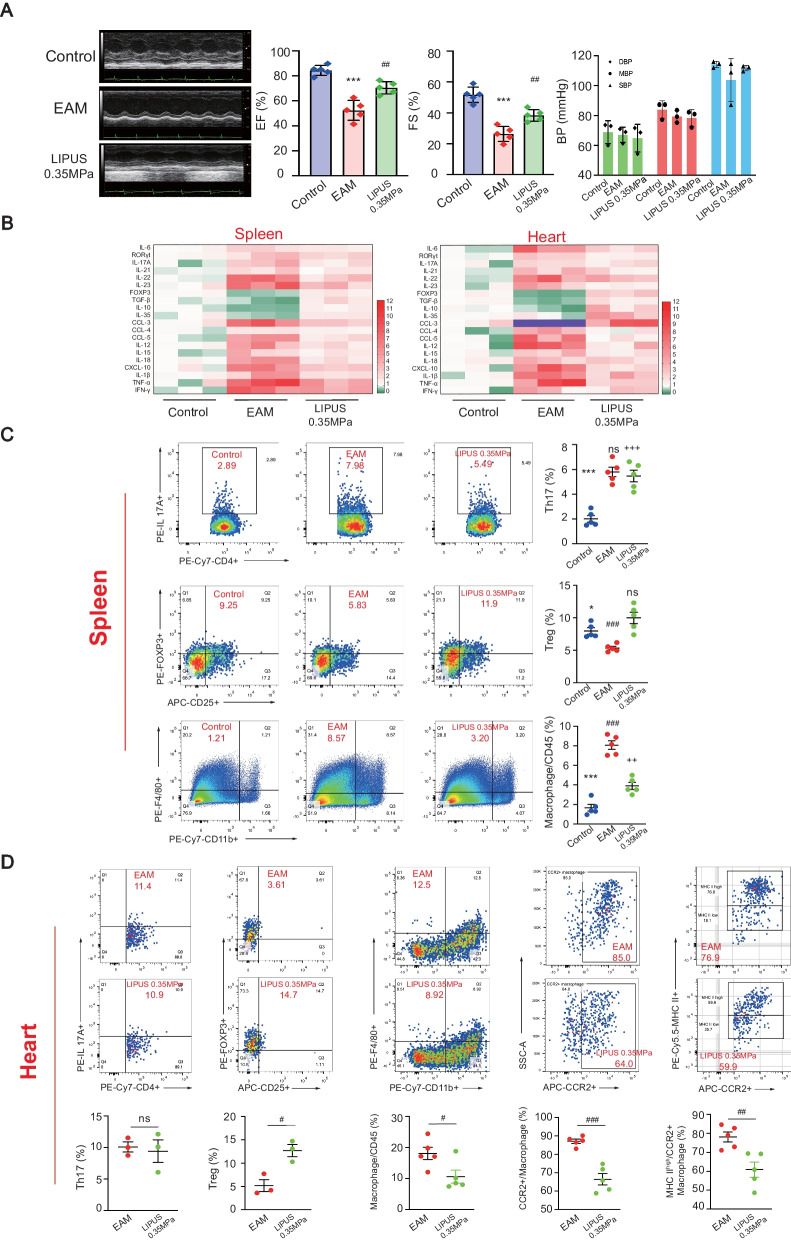
Effect of LIPUS therapy on left ventricular dysfunction and the inflammatory immune response of EAM. **A** M-mode echocardiography images, quantification of EF and FS were shown to evaluate the cardiac function of EAM mice (*n* = 5). Blood pressure showed systemic circulation under LIPUS stimulation (*n* = 3). **B** The mRNA expression of various cytokines/chemokines in spleen and heart isolated cells was detected. The expression level relative to the internal standard β-actin appeared in the heat map. **C** Representative FACS quantification of splenic Th17, Treg and macrophage from EAM mice with or without LIPUS treatment at day 21. Control mice served as a negative control (*n* = 5). **D** FACS analysis of different immune cells in heart at day 21 after EAM (*n* = 5). ^*^ Represents control vs. EAM; ^#^ represents LIPUS-0.35 MPa vs. EAM; ^+^ represents LIPUS-0.35 MPa vs. control. (Data were shown as the mean ± SD, Student’s t-test for two-group comparison and one-way analysis of variance, followed by Tukey’s multiple comparison test for comparison of multiple groups; ^*^P or ^#^*P* < 0.05, ^##^*P* or ^++^*P* < 0.01, ^***^P, ^###^*P* or ^+++^*P* < 0.001). EAM: experimental autoimmune myocarditis; EF: ejection fraction; FS: fractional shortening; LIPUS: low-intensity pulsed ultrasound; FACS: fluorescence activated cell sorting

The LIPUS treatment improved the cardiac function of EAM mice assessed on day 21 by echocardiography. In Fig. 2A and Additional file 8: Table S1, both left ventricular ejection fraction (EF) and left ventricular fractional shortening (FS) decreased in the EAM group. LIPUS ameliorated EAM-induced cardiac dysfunction with better preserved EF and FS. Due to the cardiac inflammation and injury, the heart rate of EAM mice decreased compared with the control group. LIPUS stimulation raised the heart rate from 331.70 ± 62.27 in EAM to 350.70 ± 27.48 in the US group. In addition, as demonstrated in Fig. 2A, there was no significant difference in systolic BP, mean BP, and diastolic BP among different treatment groups, indicating LIPUS targeting the spleen did not influence systemic circulation.

To evaluate the effect of LIPUS on modulating inflammatory cytokines secreted by immune cells, various inflammatory cytokines associated with Th17 cell, Treg cell and macrophage were evaluated by real-time PCR (Fig. 2B). Compared to the EAM group, the expression of Th17 maker RORγt and inflammatory cytokines IL-21, IL-23, and IL-17 in splenic samples and heart tissues were partially inhibited by LIPUS treatment. The expression of Treg maker, FOXP3 and inflammatory cytokines-IL-10, IL-35, and TGF-β in the spleen and heart were increased by LIPUS treatment. In particular, the increased inflammatory cytokines secreted by macrophages with antigen-presenting function (i.e., CCL-3, CCL-4, CCL-5, IL-12, IL-15) were inhibited by LIPUS treatment.

The CAP could regulate T cells and macrophages, which release cytokines systemically and benefit the therapeutic. As CD4+ Th17 cell/Treg cell balance and macrophages were involved in EAM pathogenesis and pathophysiology, subsets of these immune cells were analyzed by flow cytometry to determine the treatment mechanism further. First, in splenic CD4+ T cells, LIPUS treatment showed no significant benefits to decrease the percentage of CD4+ IL-17+ T cells but increased the proportion of CD25+ FOXP3+ Treg cells. The second evidence was the decreased F4/80 + /CD11b+ macrophages in the spleen of EAM mice (Fig. 2C). Furthermore, flow cytometric analysis of CD4+ T cells in isolated cardiac inflammatory cells indicated that LIPUS significantly increased the Treg cell infiltration in the myocarditis heart. In addition, we further analyzed the changes of macrophages and their subsets CCR2+ macrophage derived from and slowly replenished by circulation blood monocytes in myocarditis, and their infiltration in the heart was regarded as pathological and proinflammatory [22]. The number of immune cells isolated from healthy cardiac tissue was too low to reflect statistical difference objectively, so the percentage of cardiac immune cells was normalized to the EAM group. Compared with the EAM group, the percentage of total macrophages, especially the ratio of CCR2+ macrophage to total macrophage, decreased in the LIPUS group. The MHC II high CCR2+ macrophage ratio to CCR2+ macrophage decreased with LIPUS treatment. The results demonstrated that the migration of CCR2+ inflammatory macrophages to the myocardial and the proportion of CCR2+ MHC II high macrophage with antigen-presenting function were decreased with LIPUS treatment (Fig. 2D).

Collectively, the above data preliminarily confirmed the increased anti-inflammatory effect induced by Treg and reduced inflammatory effect induced by CCR2+ MHC II high macrophage would be involved in treating EAM by LIPUS.

### LIPUS stimulation on spleen plays a neuro-regulation role via CAP

**Fig. 3:**
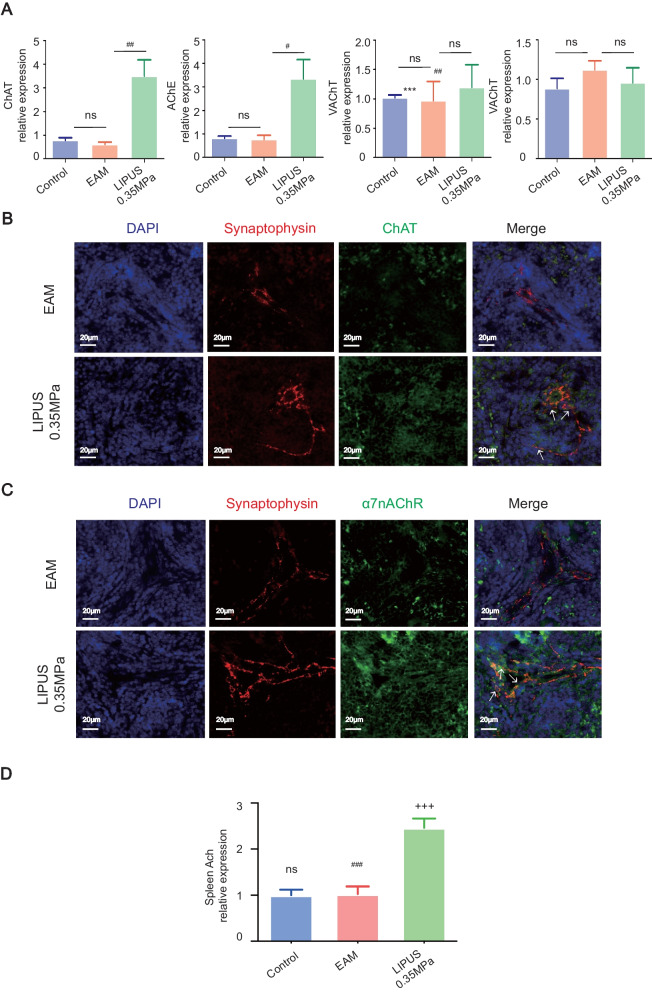
Identifying neuromodulation and cholinergic anti-inflammatory pathway involved in LIPUS therapy. **A** mRNA expression profiles of acetylcholine production-related genes in the mouse spleen. The relative abundance of transcripts was quantified and normalized to β-actin. Immunofluorescent staining detected **B** choline acetyl transferase-positive (green) or **C** α7nAChR-positive (green) cells in the spleen of EAM and LIPUS-0.35 MPa mice. Synaptophysin-positive nerve endings were found in close proximity to acetyl transferase-positive or α7nAChR-positive cells. *n* = 4. Scale: 20 μm. **D** The expression of acetylcholine in spleen of EAM mice with or without LIPUS-0.35 MPa. ^*^ Represents control vs. EAM; ^#^ represents LIPUS-0.35 MPa vs. EAM; ^+^ represents LIPUS-0.35 MPa vs. control. (Data were shown as the mean ± SD, ordinary one-way analysis of variance, Tukey's multiple comparisons test; ^#^*P* < 0.05, ^##^*P* < 0.01, ^###^*P* or ^+++^*P* < 0.001.) EAM: experimental autoimmune myocarditis; LIPUS: low-intensity pulsed ultrasound; α7nAChR: α7 nicotinic acetylcholine receptor

The spleen nerve can release norepinephrine vesicles, which act on the surrounding ACh-producing cells. The mRNA of Treg cells and macrophages were detected by real-time PCR. Enzymes associated with ACh synthesis were examined, including choline acetyltransferase (ChAT), acetylcholinesterase (ACHE), Vesicular acetylcholine transporter (VAChT) and Choline transporter 1 (CHT1). Among them, the mRNA level of ChAT and ACHE in LIPUS mice was higher than in EAM and control groups (Fig. 3A). The spleen nerve travels along the splenic artery and enters the spleen [23]. Although it is a branch of the abdominal vagus nerve, the splenic nerve is characterized by the release of NE through sympathetic fibers [23]. There is no disynaptic connection from the vagus to the spleen via the splenic sympathetic nerve and vagal stimulation does not drive action potentials in the splenic nerve [24]. Hence, ACh-synthesizing T lymphocytes provide an essential non-neural link in the anti-inflammatory pathway from the vagus to the spleen [25]. NE binds to ACh-synthesizing T lymphocytes [25] and the T cell population synthesize and release ACh under the ChAT enzyme [26]. The released ACh binds to surrounding macrophages with α7nAChR, thereby regulating the cholinergic anti-inflammatory pathway [27]. Inspired by the research published by PNAS, synaptophysin (presynaptic vesicle-associated protein) and TNF-α were stained to observe nerve endings terminate adjacent to TNF-producing macrophages in spleen [4]. Therefore, we used synaptophysin and chat double staining to locate ChAT-positive cells around nerve endings and observe the changes of cells with the ability to release ACh after LIPUS stimulation (Fig. 3B and C). The relative expression of ChAT-positive and α7nAChR-positive cell in the LIPUS group was higher than that in EAM group (Additional file 6). Additionally, the amount of ACh produced in the spleen was markedly elevated by LIPUS stimulation compared with the control or EAM group (Fig. 3D). We also measured the concentration of NE in the spleen of animals in each group. Under the treatment of different stimulated pressure, the concentration of NE in spleen indeed increased in varying degrees (Additional file 1). In conclusion, as LIPUS treatment modified immune cell subsets in EAM mice, CAP was activated in the spleen, suggesting that there may be an interaction between neuromodulation and immune response during ultrasound physical stimulus.

### The effect of LIPUS stimulation is dependent on splenic nerve

**Fig. 4:**
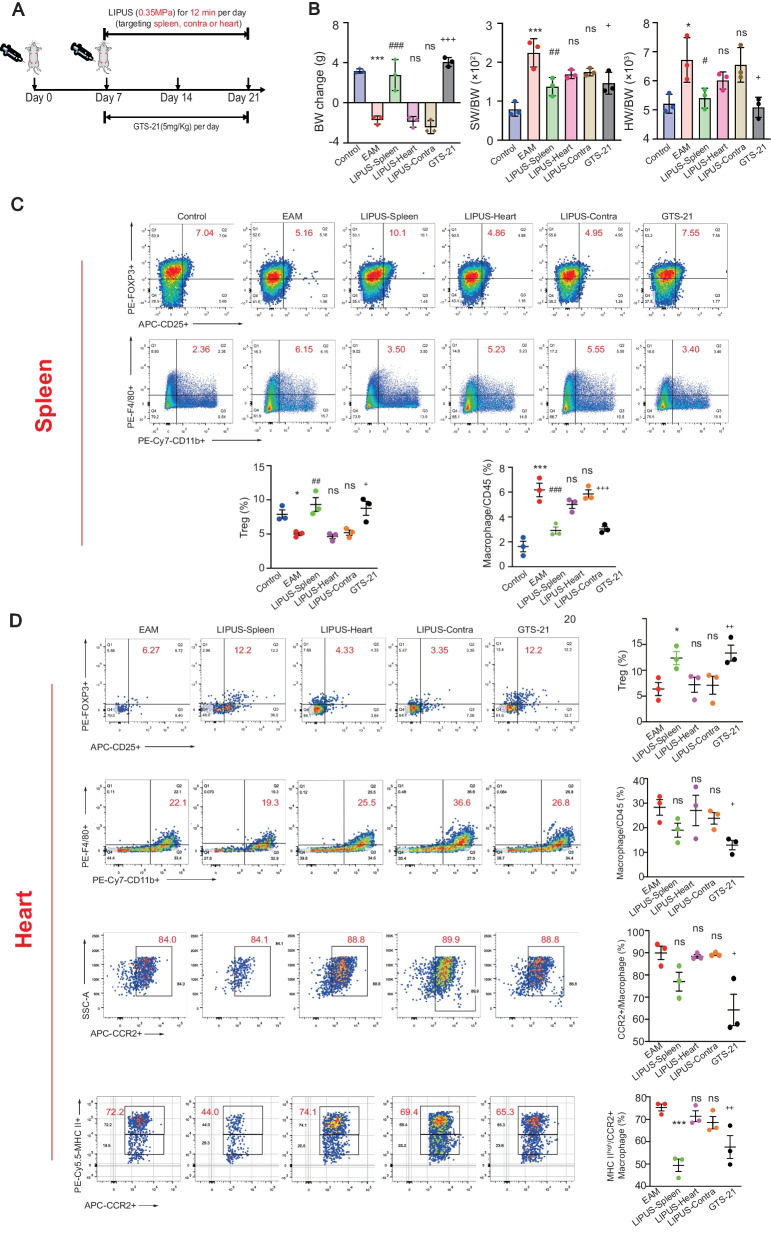
Identifying the target organ of LIPUS. **A** Timeline of LIPUS stimulation or α7nAChR agonists (GTS-21, 5 mg/kg) intervention in EAM mice. Different therapeutic targeting was assigned, including spleen, heart and contralateral abdomen opposite to the spleen. The characteristics and immune response of EAM were evaluated on day 21. **B** The BW change, SW/BW and HW/BW of EAM mice in different experimental groups (*n* = 3). **C**, **D** Representative FACS quantification of Treg and macrophage percentage of splenic (**C**) or heart (**D**) isolated immune cells from EAM mice with additional LIPUS stimulation at 21 days (*n* = 3). ^*^ Represents control vs. EAM; ^#^ represents LIPUS-spleen vs. EAM; ^&^ represents LIPUS-heart vs. EAM; ^￥^represents LIPUS-contra vs. EAM; ^+^ represents GTS-21 vs. EAM. (Data were shown as the mean ± SD, ordinary one-way analysis of variance, Tukey's multiple comparisons test; ^*^P, ^#^*P* or ^+^*P* < 0.05, ^##^*P*, or ^++^*P* < 0.01, ^***^P, ^###^*P* or ^+++^*P* < 0.001.) LIPUS: low-intensity pulsed ultrasound; α7nAChR: α7 nicotinic acetylcholine receptor; EAM: experimental autoimmune myocarditis; BW: body weight; SW/BW: spleen weight to body weight; HW/BW: heart weight to body weight; FACS: fluorescence activated cell sorting

Furthermore, to explore the dependence of ultrasound therapy on spleen nerve and α7nAChR and the optimal mode of ultrasonic application, we first investigated whether the LIPUS stimulation works in other body areas, including the heart (LIPUS-Heart) and the contralateral abdomen opposite the spleen (LIPUS-Contra) (Fig. 4A). Therefore, we used LIPUS to stimulate the abdominal position of EAM mice on the opposite side of the spleen as the control group with the same stimulation parameters and time. The LIPUS-contra group can not only avoid the surgical injury caused by selective denervation of splenic nerves in mice, but also ensure the safety and stability of EAM mice, which is conducive to the long-term observation of the dependence of LIPUS treatment on the spleen. It also helps to exclude the relevant effects of LIPUS by radiating the surrounding intestinal tract. In addition, we used an alpha7 nicotinic ACh receptor (α7nAChR) agonist to compare with the LIPUS treatment. GTS-21 dihydrochloride is a selective α7nAChR agonist with CAP anti‑inflammatory activities, which has been reported to alleviate acute lung injury [28] and cisplatin-induced nephropathy [29]. In our study, the LIPUS targeted spleen stimulation showed a similar trend as the GTS-21 treatment (positive control) by evaluating the essential characteristics of myocarditis (Fig. 4B) and flow cytometric analysis of Treg and macrophages in the spleen and cardiac tissue (Fig. 4C and D). However, the efficacy of cardiac-target and contra-spleen LIPUS treatment was not as good as that of the optimal parameters, which could alleviate the immune response and inflammatory injury of the EAM heart. We predicted from the above results that the spleen was the suitable organ to target during LIPUS treatment.

**Fig. 5:**
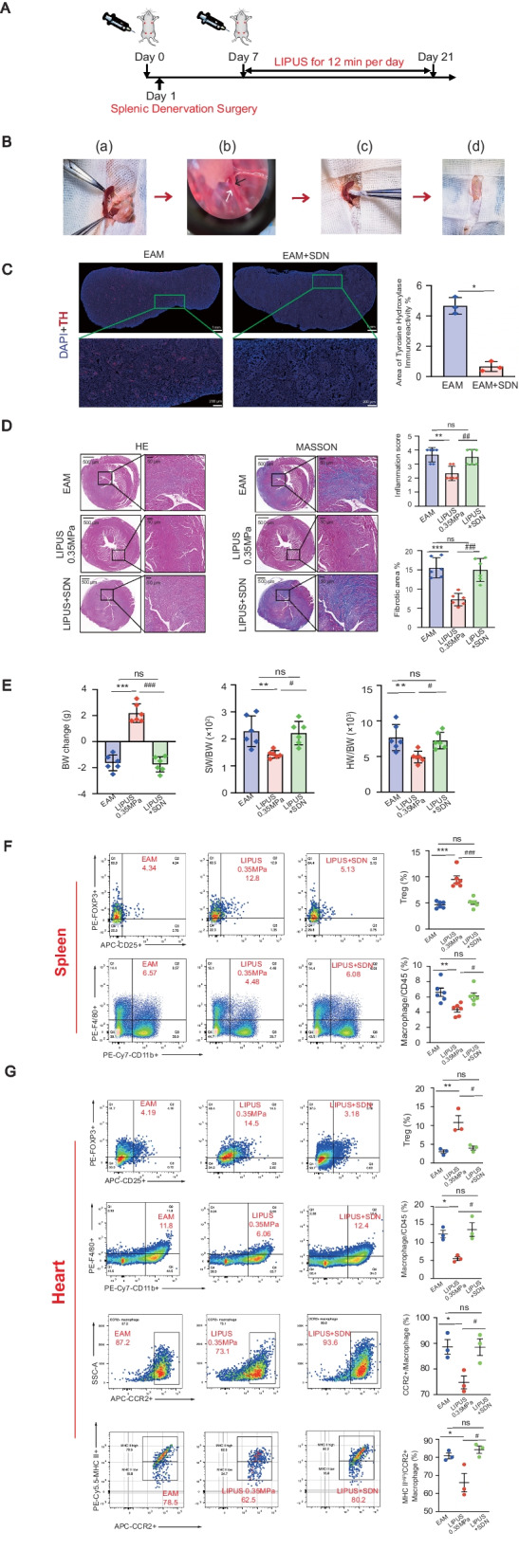
The effect of LIPUS stimulation on EAM mice with splenic denervation surgery. **A** Timeline of a LIPUS treatment experiment on EAM mice with splenic denervation surgery. **B** Photographs of surgical exposure and alcohol application for denervation operation. **C** Representative images of whole spleen sections of EAM mice or denervated EAM mice 6 days after surgery. Red, tyrosine hydroxylase (TH) staining. Blue, nuclear staining. In statistical analysis, quantification revealed a significant decrease in tyrosine hydroxylase immunoreactivity in denervated spleens relative to spleens from mice subjected to EAM (*n* = 3). ^*^ Represents EAM vs. EAM + SDN. **D** HE and MASSON staining for the degree of inflammatory cell infiltration and collagen deposition of EAM hearts. Scale: 500 μm (top) and 50 μm (bottom). Representative HE and MASSON staining images of indicated groups. Bar graphs on the right show quantitation of data (*n* = 6). **E** The BW change, SW/BW and HW/BW of EAM mice in different experimental groups (*n* = 6). **F**, **G** Representative FACS quantification of Treg and macrophage percentage of splenic (**F**) or heart (**G**) isolated immune cells from EAM mice with additional LIPUS stimulation at 21 days (*n* = 6). ^*^ Represents EAM vs. LIPUS-0.35 MPa; ^#^ represents LIPUS-0.35 MPa vs. LIPUS + SDN; ^+^ represents EAM vs. LIPUS + SDN. (Data were shown as the mean ± SD, Student’s t-test for two-group comparison and one-way analysis of variance, followed by Tukey’s multiple comparison tests for comparison of multiple groups; ^*^P or ^#^*P* < 0.05, ^**^P or ^##^*P* < 0.01, ^***^P or ^###^*P* < 0.001.) EAM: experimental autoimmune myocarditis; LIPUS: low-intensity pulsed ultrasound. SDN: splenic denervation; TH: tyrosine hydroxylase; HE: hematoxylin and eosin; BW: body weight; SW/BW: spleen weight to body weight; HW/BW: heart weight to body weight; FACS: fluorescence activated cell sorting

In addition, we verified the splenic nerve dependence of LIPUS in the treatment of myocarditis. The timeline of LIPUS treatment experiment on EAM mice with splenic denervation surgery is shown as Fig. 5A. The surgery of splenic denervation (SDN) was performed in EAM mice (Fig. 5B). Since the splenic nerve denervation operation only inactivated most of the splenic nerve fibers and did not harm the spleen vessels, HE and CD31 staining of the spleen demonstrated that the histology and the blood supply in the spleen was normal (Additional file 7). LIPUS reduced myocardial inflammatory damage in EAM mice, cardiac inflammation (HE staining and MASSON staining as shown in 5D), as indicated by changes in general features (Fig. 5E), and immune cell subsets of spleens and hearts (Fig. 5F, G). In contrast, in the above indexes, LIPUS treatment of mice with denervation of splenic nerve did not show statistically significant differences compared with the EAM group alone. The therapeutic effect of LIPUS on mice with denervation of splenic nerve was not as good as that on mice with intact splenic nerve.

### RNA sequencing in LIPUS-treated animals

**Fig. 6:**
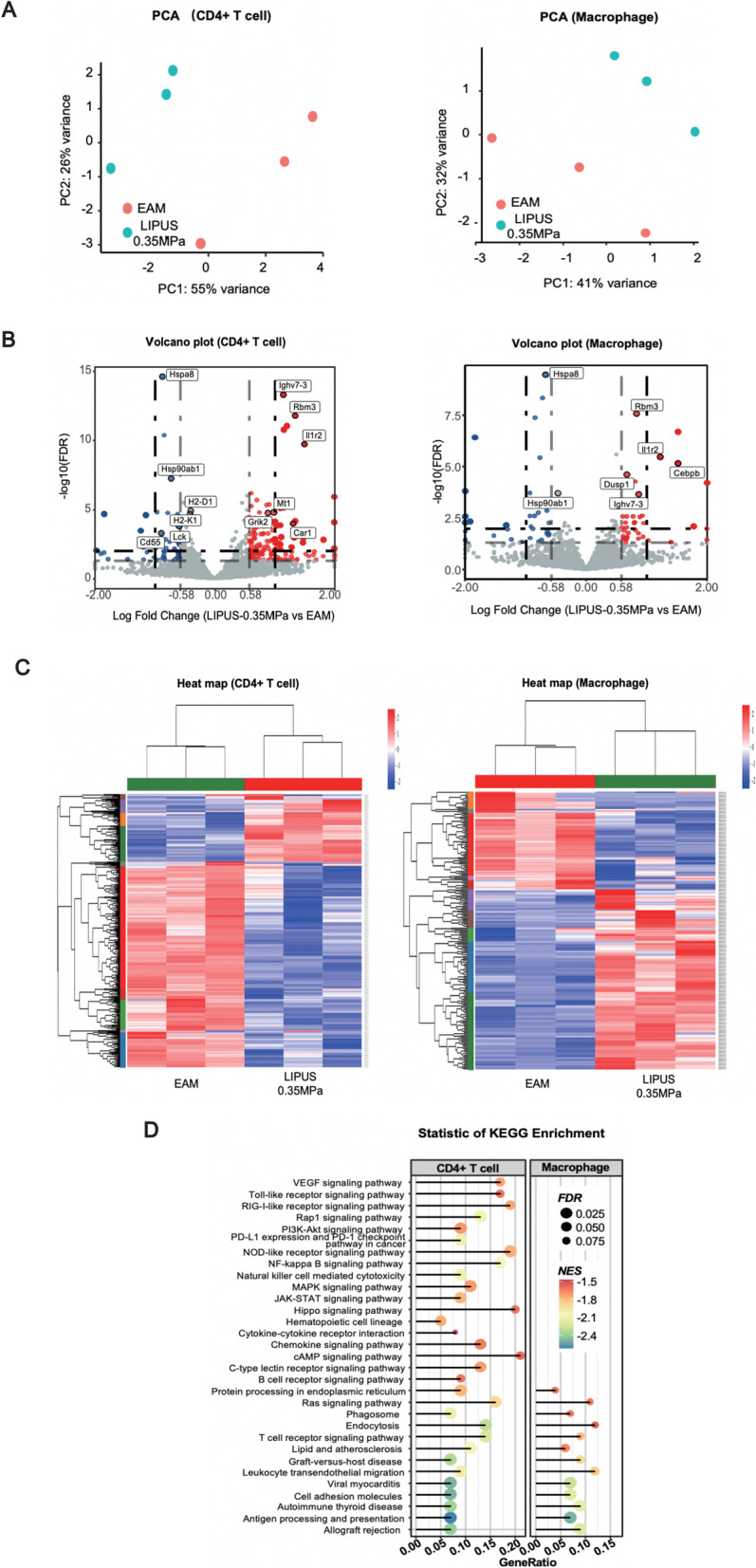
RNA sequencing of T cell and macrophage in LIPUS-treated animals. **A** Two-dimensional PCA representation of CD4+ T cell and CD11b + macrophage transcriptomes at day 21. **B** Volcano plot of DEGs from T cell and macrophage between EAM and LIPUS-0.35 MPa mice. Log fold change was plotted against the Edge R-generated *p*-value (−log base 10). Red indicates upregulated genes, and blue indicates downregulated genes. The gray area shows the gene expression below the threshold criteria (|log2fold-change|> 0.58 or adjusted *P*-value < 0.05 or *P*-value < 0.01). **C** Hierarchical clustering of DEGs in CD4+ T cell and CD11b+ macrophage of EAM group and LIPUS stimulation group. **D** Pathway analysis of significantly enriched DEGs. The top 31 statistically significant enriched pathways are shown. LIPUS: low-intensity pulsed ultrasound; EAM: experimental autoimmune myocarditis; PCA: principal component analysis; DEGs differential expressed genes

The above results demonstrated that the efficacy of LIPUS depends on specific targeting of the spleen. We sought to determine the molecular mechanisms underlying the reduced cardiac inflammation seen with LIPUS treatment by investigating the gene expression profiles of CD4+ T cell and CD11b+ macrophage in the spleen after LIPUS therapy using transcriptome sequencing. We compared EAM mice with or without Spleen-targeted LIPUS treatment (*n* = 12, three animals in each group). Principal component analysis (PCA) and heat-map data showed differential expressed genes (DEGs) between the EAM and LIPUS groups. The PCA may provide clues about the biological effects of targeted spleen ultrasound in myocarditis (Fig. 6A). Among these genes of T cell or macrophage, gene expression was normalized across samples to percentages ranging from marked upregulation (deep red) to marked downregulation (deep blue) (Fig. 6C). Differential expression analysis of T cells using a *P* < 0.05 or *P* < 0.01 cutoff identified 75 DEGs or 49 DEGs between EAM and LIPUS mice. The gene list of macrophages was cut to 17 and 13 DEGs using cutoffs of *P* < 0.05 or *P* < 0.01, respectively. Among these genes, 5 DEGs were identified in both T and macrophage cells in the LIPUS stimulation group, including Hspa8, Rbm3, Il1r2, Hsp90ab1 and Ighv7-3, suggesting that LIPUS induces overlapping effects in these two cell types. Additionally, DEGs of T cells were focused on immune cell differentiation, metabolism and antigen presentation (Fig. 6B). KEGG pathway significant enrichment analysis of DEG function was also performed to identify the main biochemical metabolic pathways and signal transduction pathways. Results indicated that significantly enriched pathways included antigen processing and presentation, JAK–STAT signaling pathway, C-type lectin receptor signaling pathway, cell adhesion molecules, endocytosis and immune-associated pathway. The transcriptome sequencing data determined the DEGs due to LIPUS stimulation (Fig. 6D).

### The LIPUS stimulation also has therapeutic effect on chronic EAM mice

**Fig. 7:**
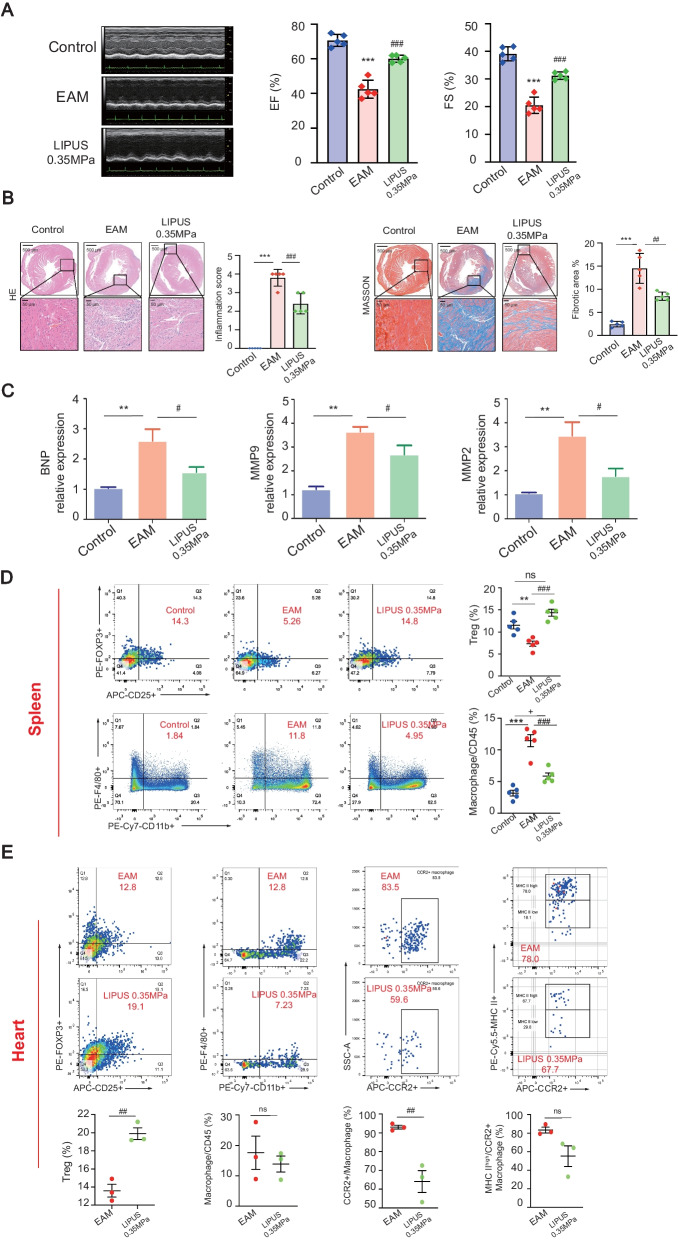
The effect of LIPUS stimulation on immune response of chronic EAM mice. **A** M-mode echocardiography images, quantification of EF and FS were shown to evaluate the cardiac function of EAM mice (*n* = 5). **B** HE and MASSON staining for the degree of inflammatory cell infiltration and collagen deposition of EAM hearts. Scale: 500 μm (top) and 50 μm (bottom). Representative HE and MASSON staining images of indicated groups. Bar graphs on the right show quantitation of data (*n* = 5). **C** The expression of BNP, MMP2 and MMP9 in heart of EAM mice with or without LIPUS-0.35 MPa (*n* = 3). Representative FACS quantification of Treg and macrophage percentage of splenic (**D**) or heart (**E**) isolated immune cells from EAM mice under LIPUS-0.35 MPa stimulation until day 56 (*n* = 5). ^*^ Represents control vs. EAM; ^#^ represents LIPUS-0.35 MPa vs. EAM; ^+^ represents LIPUS-0.35 MPa vs. control. (Data were shown as the mean ± SD, Student’s t-test for two-group comparison and one-way analysis of variance, followed by Tukey’s multiple comparison test for comparison of multiple groups; ^#^*P* or ^+^*P* < 0.05, ^**^P or ^##^*P* < 0.01, ^***^P or ^###^*P* < 0.001.) LIPUS: low-intensity pulsed ultrasound; EAM: experimental autoimmune myocarditis; EF: ejection fraction; FS: fractional shortening; HE: hematoxylin and eosin; FACS: fluorescence activated cell sorting; MMP2: matrix metallopeptidase 2; MMP9: matrix metallopeptidase 9; BNP: B-type natriuretic peptide

To investigate the potential to inhibit cardiac remodeling by LIPUS therapy via CAP in chronic EAM mice, we extended the experimental observation period to 56 days. As shown in Fig. 7A and Additional file 8: Table S2, the mice in the EAM group suffered worse left ventricular dysfunction with a significant decrease in EF and FS. LIPUS stimulation ameliorated EAM-induced cardiac dysfunction with better preserved EF and FS. In Fig. 7B, HE-stained EAM heart sections demonstrated a high inflammation score, which was reduced by LIPUS treatment. Myocardial fibrosis was reduced by LIPUS treatment as indicated by reduced collagen deposits in treated groups. At the molecular level in Fig. 7C, the increased mRNA expression of BNP could be inhibited by LIPUS stimulation, indicating that LIPUS could improve cardiac insufficiency in myocarditis. Similar trends were observed in the mRNA expression of Matrix Metallopeptidase 2 (MMP2) and MMP9, which is consistent with the histological observations above, showing that LIPUS therapy would significantly ameliorate the inflammation-induced cardiac fibrosis. The flow cytometric analysis in Fig. 7D and E demonstrated that the Treg cell and macrophages isolated from the spleen and heart on day 56 were similar to day 21. LIPUS treatment significantly increased the proportion of Treg cells in both the spleen and heart. In contrast, a decreased total macrophage portion was found in the spleen. Although there was no significant decrease in total macrophages in the heart, the CCR2+ macrophage group and its MCH II subsets showed a corresponding change, lowering the inflammation response.

## Discussion

Therapeutic strategies of EAM that can modulate CD4+ Th17/Treg immune responses, inhibit cytokine-producing from macrophages, and restrain migration of activated macrophages into the myocardium have been reported to show benefits in ameliorating cardiac inflammation and reducing myocardial injury [30]. Considering the long-term legacy of inflammatory damage to myocarditis caused by immune imbalance and the lack of a cure that patients have a poor quality of life, the noninvasive portable treatment is an available option. In this study, LIPUS was used to stimulate the spleen nerve and alleviate autoimmune myocarditis. We evaluated the dependence of the spleen and cholinergic anti-inflammatory pathway in treating autoimmune myocarditis with LIPUS in mice. It revealed that splenic ultrasound could alleviate the immune response, regulate the proportion and function of CD4+ Treg and macrophages by activating CAP, and finally reduce heart inflammatory injury and improve cardiac remodeling. While investigating the therapeutic efficacy and optimizing the therapeutic parameters for EAM with LIPUS, the targeted organ and enough acoustic pressure have played an important role in EAM treatment.

### (1) The spleen is suitable for ultrasound stimulation

Firstly, we determined the center of the spleen by following the research published in *Nature Communication* by Zachs et al., which shows that the midpoint of the connecting line from the shoulder and hip joints of mice is considered the spleen’s location [16]. And we measured the thickness and depth of the spleen of normal BALB/c mice (width * thickness: 4.84 mm*2.02 mm) and EAM mice (width * thickness: 5.77 mm * 2.48 mm) used in this study (Additional file 2). The ultrasound transducer’s sound field was measured using a needle hydrophone (ONDA HNA-0400) to clarify the transducer’s sound pressure distribution. As shown in Additional file 4, from the tip of the coupling cone to the other end of the ultrasound focus, the long axis of the ultrasound focus is about 4 mm (red line). Therefore, the ultrasound focal depth is comparable to the depth of the spleen from the skin, which can be sonicated properly. In summary, we use an external transducer to emit focused ultrasound waves that penetrate the skin to a certain depth to work on the spleen. This method does not guarantee that it only works on the spleen and avoids all other organs. However, according to the above explanation and the ultrasound foci size and depth, the ultrasound wave cannot deliver enough sound energy to stimulate the surrounding tissues, so it should have little impact on the surrounding intestinal tract and celiac plexus. Moreover, the LIPUS-Contra group, shown in Fig. 4, is ultrasound stimulation of the contralateral intestinal tract of the spleen, which has no significant therapeutic effect on EAM. To some extent, it is proved that the LIPUS stimulus caused therapeutic effects through the spleen rather than the surrounding intestinal tissues and celiac nerves.

### (2) The therapeutic effect of ultrasound stimulation depends on acoustic pressure

In the study of Cotero et al., splenic NE levels averaged 140 nmol/L in rodents (wild-type C57black/6 mice or Sprague-Dawley rats), whereas the LPS group dropped NE levels to near zero, demonstrating suppression of CAP signaling during LPS-induced inflammation [15]. With the gradual increase of acoustic pressure, the concentration of NE from spleen increases first and then decreases in ultrasound-stimulated mice. At 0.25 MPa, the average NE concentration reached its peak. In our Additional file 1, under the treatment of different stimulated pressure, the concentration of NE in spleen indeed increased in varying degrees. It suggested that the response of NE to the ultrasound is dependent on stimulated pressure. However, under the effect of 0.3 MPa and 0.473 MPa, there was no significant difference in NE level. Similarly, there was no significant difference in the therapeutic effect between the 0.35 MPa and 0.473 MPa acoustic pressure as shown in Figs. 1, 2. There may be an acoustic pressure threshold for neuronal activation to release neurotransmitters and produce neuromodulation effects. In addition, the LIPUS stimulation duration of each treatment was another determinant, which was even more important than the total treatment length across the LIPUS experiment. Spleen stimulation for 12 min at 0.35 MPa was more effective at reducing myocarditis than 6 min, and the optimized parameters were consistently effective in subsequent experiments. Combining our results with previous studies, we speculated that the production of neurotransmitters is an accumulation process that requires adequate stimulation time.

### (3) The selection of vagus nerve segment for ultrasound stimulation

The vagus nerve is the main nerve of the parasympathetic division of the autonomic nervous system. Its efferent arm is distributed in major body organs, including the heart, liver, digestive system and spleen [31]. The efferent vagal nerve-mediated cholinergic signal controls the immune function and proinflammatory response through inflammatory reflex. The intervention of it can produce an anti-inflammatory reaction and have a therapeutic effect on sepsis, renal ischemia, colitis, arthritis, etc. [32–35] Compared with the systemic regulation by oral drugs, physical stimulation of a certain vagus nerve segment can avoid dysfunction of other important tissues and organs caused by activation of the vagus nerve in the whole body. In previous studies, most of the physical stimulation sites of vagus nerve were selected to be controlled by cervical vagus nerve [36–38], apical splenic nerve [39] or celiac ganglion [40–42]. Electrical stimulation is the most common form of physical therapy. Implantation of electrodes and generation of different voltages to stimulate nerves are the main intervention methods, but there are some side effects, such as dyspnea, pain and cough. Stimulation of cervical vagus nerve causes the excitation of branches of cardiac afferent nerve, leading to a long-term decrease in heart rate and increased heart rate variability. In addition, since electrodes need to be implanted surgically, many postoperative complications may occur, such as bleeding, infection, permanent vocal cord paralysis, coma, etc. [43, 44]. Therefore, it is of great significance to find a noninvasive means of vagus nerve regulation, which is also the purpose of this study. We choose the spleen, one of the vagus nerve branch, as the stimulated organ. The spleen is an important immune organ of the human body, and is an indispensable part for the activation, development, differentiation and maturation of a variety of immune cells. Previous studies have shown that the spleen can be used as the cross-talk bridge between nerve and immunity, and that the cholinergic anti-inflammatory pathway of the splenic nerve can be used to regulate the differentiation and response of T cells and macrophages [45, 46]. This intervention approach will not affect the normal operation of other organs, especially to avoid the risk of inhibiting heart rate and arrhythmia, which is very important for cardiovascular disease treatment. The denervation of splenic nerve surgery was performed in EAM mice in Fig. 5. LIPUS treatment of mice with denervation of splenic nerve did not show statistically significant differences compared with the EAM group alone. The therapeutic effect of LIPUS on mice with denervation of splenic nerve was not as good as that on mice with intact splenic nerve. Since the splenic nerve denervation operation only inactivated most of the splenic nerve fibers, the spleen vessels were not harmed and the blood supply in the spleen was normal, and the immune cells were still alive but not regulated by the splenic nerve. Therefore, the comparison between LIPUS + EAM group and LIPUS + EAM + denervation of splenic nerve group at least partially explains the splenic nerve dependence of the ultrasound treatment in this study.

In the progress of EAM, the proliferative or imbalance of CD4+ Th17/Treg and activated macrophages play a prominent role, contributing to the severity of EAM inflammation [22, 47, 48]. Therefore, the dynamic change of different immune cell subsets is one of our research's focuses, contributing to the further understanding of myocarditis pathogenesis. We found that the modulatory effect of LIPUS stimulation was more evident on Treg than Th17 cells. The reduction of inflammatory CCR2+ macrophages with antigen-presenting function and the increase of immune-tolerant Treg cells work synergistically in LIPUS immune process control. Among the CCR2+ macrophage, the proportion of high MHC II expression in F480+/CD11b+ CCR2+ positive macrophages decreased significantly after LIPUS treatment. As MHC II represents the antigen presentation ability to T cells, we speculated that the CCR2+ macrophages with MHC II high expression showed the function of antigen-presenting cells to regulate the Treg immune response. Since the classification of M1/M2 macrophages has been replaced gradually due to many overlapping functions and markers [48], the blood-derived CCR2+ macrophages, which are involved in cardiac dysfunction and fibrosis, inhibited oxidative stress, myocardial apoptosis and cardiac inflammation, have been used to distinguish between proinflammatory and cardiac resident macrophage [49, 50]. Leuschner reported that mice silenced CCR2 exhibit a reduced inflammation in autoimmune myocarditis [51]. In addition, transcriptome sequencing results showed that DEGs were associated with T cell differentiation, metabolism and antigen presentation and involved in oxidative stress response. Taken together, both adaptive and innate immunity is involved in the biological mechanism underlying LIPUS treatment.

Noninvasive ultrasound stimulation is demonstrated to be effective in treating EAM at both the organ level and the cellular and molecular levels. We also determined that ultrasound treatment in EAM mice activates the splenic nerve. As an essential signaling pathway for neuro-immune modulation, the CAP of the spleen provides a crucial bridge for LIPUS to exert cardiac protective effects. Stimulating other body areas such as the heart or the contralateral abdomen opposite the spleen did not effectively improve myocarditis. Hence, the positive results of LIPUS therapy appeared to be not mediated by a direct effect on the heart but immunomodulation via CAP that is dependent upon the spleen. Moreover, ultrasound stimulation showed similar therapeutic efficacy as the α7nAChR agonists, GTS-21, which decreased heart inflammation in a murine virus and autoimmune myocarditis model [53, 54]. Moreover, right cervical vagotomy inhibited the CAP, aggravated myocardial lesions, and upregulated the expression of TNF-α, IL-1β, and IL-6. It worsened the impaired left ventricular function in murine viral myocarditis, and these changes were reversed by co-treatment with nicotine by activating CAP [55]. Therefore, the ultrasound stimulation can function via CAP like vagal stimulation, which has been demonstrated to improve the survival rate and prevent the progression of cardiac dysfunction and remodeling [56]. Considering the insidious onset and rapid progress of myocarditis, LIPUS may not participate in the clinical treatment in the acute phase timely, but applying it in the convalescence stage is encouraging. Given that LIPUS is used in the clinic, this adds a valuable treatment option for patients with EAM. With the advantage of safety, availability and portability of LIPUS, it could be worn by patients over their abdominal area, significantly improving their quality of life and demonstrating a perspective of clinical transformation. There is an emerging need for noninvasive neuromodulation techniques to minimize adverse events and morbidity during improving patient outcomes. The intensity of ultrasound used in the LIPUS therapy is below the upper limit of acoustic output standards for diagnostic ultrasound devices, making it suitable for home care during recovery. For the first time, LIPUS has become an adjuvant therapy for EAM.

## Limitation

It has been reported that CCR2+ MHC-II high has a stronger ability to attract other inflammatory cells, such as monocytes expressing different molecules [22]. In myocarditis induced by Coxsackievirus B3, CX3CR1−/− CVB3 mice suffered an increase in immune cell infiltration in the myocardium. This shift towards a proinflammatory immune response further resulted in increased cardiac fibrosis and cardiomyocyte apoptosis, which is a process that is activated after virus infection in acute myocarditis [52]. However, there is no similar study on autoimmune myocarditis. In Additional file 5, there was no significant difference in immunohistochemical staining of CX3CR1 in myocardial tissue among all groups. In addition, in Fig. 2B, due to the diversity of inflammatory factors released by macrophages, we detected a variety of related cytokines, including IL-12, IL-15, CCL-5, TNF-a, etc. Since these inflammatory factors have changed to varying degrees, it could not be further determined which kind of monocyte is more important for CCR2+ macrophage chemotaxis. In the LIPUS treatment of EAM, the response of chemotactic cell subsets of CCR2+ macrophages need to be confirmed by more work in future studies.

In addition, α7nAChR antagonist was not chosen to prove the dependence of LIPUS on α7nAChR in the treatment of myocarditis. At present, the α7nAChR antagonist used in the study of mouse cardiovascular disease model is methyllycaconitine citrate (MLA), which is rarely used. We found that MLA promoted the death of mice with hemorrhagic shock, accompanied by a continuous decrease in hemoglobin content, and a significant increase in the dry and wet weight of the heart [57]. In another recent study, MLA, as a control group, caused myocardial reperfusion injury in mice after cardiopulmonary resuscitation and continued to reduce cardiac function [58]. More importantly, both hemorrhagic shock and cardiopulmonary resuscitation are acute models that are generally observed for only 3 or 72 h. The cardiotoxicity of MLA is unknown. However, EAM mice need to take oral MLA daily to antagonize α7nAChR for 21 days continuously. The risk of heart injury is very high, which is not conducive to the survival of mice and the observation of the therapeutic effect of LIPUS. Selective denervation of splenic nerves was not chosen as the control group. It is a life-threatening and invasive operation. Since the splenic nerve is extremely thin and closely connected with the splenic artery, it is very easy to damage the artery during the operation, leading to massive hemorrhage and death of mice. Therefore, we do not choose α7nAChR antagonist and selective denervation of splenic nerves as the control.

Moreover, ultrasound has received more and more attention in the past decade due to its ability to regulate neuronal activities non-invasively. The low-intensity ultrasound could reversibly regulate the physiological activities of neurons in peripheral nerves, the spinal cord and complete brain circuits [59]. The experimental evidence shows that the sound pressure exerted by ultrasound acts on mechanically sensitive ion channels to a certain extent to regulate their activity. And various types of ion channels may be involved, such as calcium-selective mechanosensitive ion channel [60, 61], Transient Receptor Potential A1 [62], mechanosensitive K + channel TRAAK [63], mechanosensitive ion channel PIEZO2 [64] and so on. These all depend on the bio-mechanical effects of ultrasound, which the acoustic pressure of ultrasound could cause change cell bilayer membrane conductance and ion channel activity [56]. However, the precise mechanisms of action enabling ultrasound to both stimulate and suppress neuronal activity remain to be clarified. This is the limitation of this paper and also the direction of our future exploration of the mechanism of ultrasound as a means of physical therapy.

## Conclusions

In conclusion, our studies have found that, as a non-pharmacological and safety technique, therapeutic ultrasound was shown here to change the immunological profile of an EAM model in mice, leading to clinical and pathological changes that attenuated myocardial inflammatory damage and remodeling. LIPUS can promote Ach production cells to release acetylcholine, activate the CAP, and regulate the function of different immune cells, especially CD4+ Treg and CCR2+ macrophages, and thus inhibit their infiltration into cardiac tissue, so as to alleviate the acute myocardial injury in EAM mice and play a positive role in long-term heart recovery by stimulating splenic nerves.

## Supplementary Information

**Additional file 1.** The expression of NE in spleen of EAM mice under stimulation of different acoustic pressure of LIPUS (*n*=5). ns represents control vs. EAM or LIPUS-0.1 MPa vs. EAM;^*^ represents LIPUS-0.35 MPa vs. EAM; ^+^ represents LIPUS-0.473 MPa vs. EAM. (Data were shown as the mean ± SD, ordinary one-way analysis of variance, Tukey's multiple comparisons test; ^###^*P* or ^+++^*P* < 0.001) *EAM: Experimental Autoimmune Myocarditis; NE: norepinephrine; LIPUS: low-intensity pulsed ultrasound.*

**Additional file 2.** LIPUS stimulated localization on the spleen and measurement of mouse spleen dimensions. Mice were anesthetized with isoflurane before LIPUS stimulation. Since the spleen is shallow, it can be clearly outlined and marked through the thin skin (A-B). To confirm the spleen's location, the mouse skin was turned over (C). The spleen is consistently located halfway between the shoulder and hip joints of the animal, so the landmarks can be used to target the spleen (D). The depth (E), length (F), width (G) and thickness (H) of spleen were measured after euthanasia. These three-dimension data are recorded in mm in Table I (bottom). (*n*=5). *LIPUS: low-intensity pulsed ultrasound.*

**Additional file 3.** Cardiac and splenic immune cell subsets gating strategy. (A) Splenic T cell and macrophage gating strategy. (B) Cardiac T cell and macrophage gating strategy. *FACS: Fluorescence Activated Cell Sorting.*

**Additional file 4.** Sound field scanning of focused ultrasound transducer. (A) The concave transducer (OLYMPUS V302) with a custom-designed focusing cone (B) filled with agarose gel (1.5%, w/v). The ultrasound transducer’s sound field showed three-dimensional focused area (C) and focal depth (D). A black line shows the edge of the coupling cone; this is the level of contact with the skin. The focused sound field is distributed within 4mm depth (between black and white line). The system (E) consists of a Function Generator (Tektronix AFG3051C), a power amplifier (AG 1020, T&C Power Conversion, Inc.) and a concave transducer (OLYMPUS V302).

**Additional file 5.** Effect of LIPUS on the immunohistochemical staining of CX3CR1 in myocardium. Representative immunohistochemical staining of CX3CR1 in myocardium of control, EAM, and LIPUS group (*n*=4). Scale bar represents 50 μm. *EAM: Experimental Autoimmune Myocarditis; LIPUS: low-intensity pulsed ultrasound.*

**Additional file 6.** Nerve endings terminate adjacent to ChAT-positive or α7nAChR-positive cell in spleen. Synaptophysin-positive (green) nerve endings were found in close proximity to ChAT-positive (A: red) or α7nAChR-positive (B: red) in LIPUS group of mice (especially white arrow). In statistical analysis, the number of target cell in LIPUS group is normalized to EAM group (bottom). (*n*=4) ^*^ represents LIPUS-0.35 MPa vs. EAM. (Data were shown as the mean ± SD, unpaired T test, Mann-Whitney test; ^*^P < 0.05) *EAM: Experimental Autoimmune Myocarditis; LIPUS: low-intensity pulsed ultrasound; ChAT: choline acetyl transferase; α7nAChR: alpha7 nicotinic ACh receptor.*

**Additional file 7.** The effect of splenic denervation on vessels of EAM mice. A. HE staining for spleen histological signs of EAM mice and EAM+SDN mice. B. Representative images of whole spleen sections of EAM mice and EAM+SDN mice. Blue, nuclear staining. Green, CD31 is a sensitive and specific marker for vascular endothelial injury (especially white arrow). *EAM: Experimental Autoimmune Myocarditis; SDN: Splenic Denervation; HE: hematoxylin and eosin.*

**Additional file 8. Table S1**. Echocardiographic analysis of control, EAM and LIPUS treated mice groups on day 21. Summary of heart rate, ejection fraction, fractional shortening, left ventricular internal dimension diastolic (LVID diastolic), left ventricular internal dimension systolic (LVID systolic), left ventricular end-diastolic volume (LVEDV) and left ventricular end- systolic volume (LVESV) of health control mice, EAM mice and LIPUS mice after 21 days’ model, respectively. Data are presented as mean ± SD; ^**^ *P < *0.01; ^***^ *P < *0.001 versus Control; ^#^ *P < *0.05; ^##^ *P < *0.01; ^###^ *P < *0.001 versus EAM; ^†^ *P < *0.05; ^††^ *P < *0.01; ^†††^ *P < *0.001 versus Control. **Table S2**. Echocardiographic analysis of control, EAM and LIPUS treated mice groups on day 56. Summary of heart rate, ejection fraction, fractional shortening, left ventricular internal dimension diastolic (LVID diastolic), left ventricular internal dimension systolic (LVID systolic), left ventricular end-diastolic volume (LVEDV) and left ventricular end- systolic volume (LVESV) of health control mice, EAM mice and LIPUS mice after 56 days model, respectively. Data are presented as mean ± SD; ^***^ *P < *0.001 versus Control; ^##^ *P < *0.01; ^###^*P < *0.001 versus EAM; ^††^ *P < *0.01; ^†††^ *P < *0.001 versus Control. Data are presented as mean ± SD. **Table S3**. Sequences of the primers for real time RT-PCR. Table S4 FACS Information of splenic and cardiac immune cells.

## Data Availability

All data generated or analyzed during this study are included in this published article and its supplementary information files.

## Abbreviations

CAP: Cholinergic anti-inflammatory pathway
LIPUS: Low-intensity pulsed ultrasound
ACh: Acetylcholine
ChAT: Choline acetyltransferase
VNS: Vagus nerve stimulation
US: Ultrasound
BW: Body weight
SW/BW: Spleen weight to body weight
HW/BW: Heart weight to body weight ratio
EF: Ejection fraction
FS: Fractional shortening
CFA: Complete Freund’s adjuvant
EAM: Experimental autoimmune myocarditis
H&E: Hematoxylin and eosin
ELISA: Enzyme-linked immunosorbent assay
BP: Blood pressure
LPS: Lipopolysaccharide
TPM: Transcripts per million
FDR: False Discovery Rate
GSEA: Gene Set Enrichment Analysis
MLA: Methyllycaconitine citrate
ACHE: Acetylcholinesterase
VAChT: Vesicular acetylcholine transporter
CHT1: Choline transporter 1
