# Supplementary figures and images for: Noninvasive ultrasound stimulation to treat myocarditis through splenic neuro-immune regulation
Source: J Neuroinflammation. 2023 Apr 17;20:94. doi: 10.1186/s12974-023-02773-2 (PMC10108488; doi:10.1186/s12974-023-02773-2)

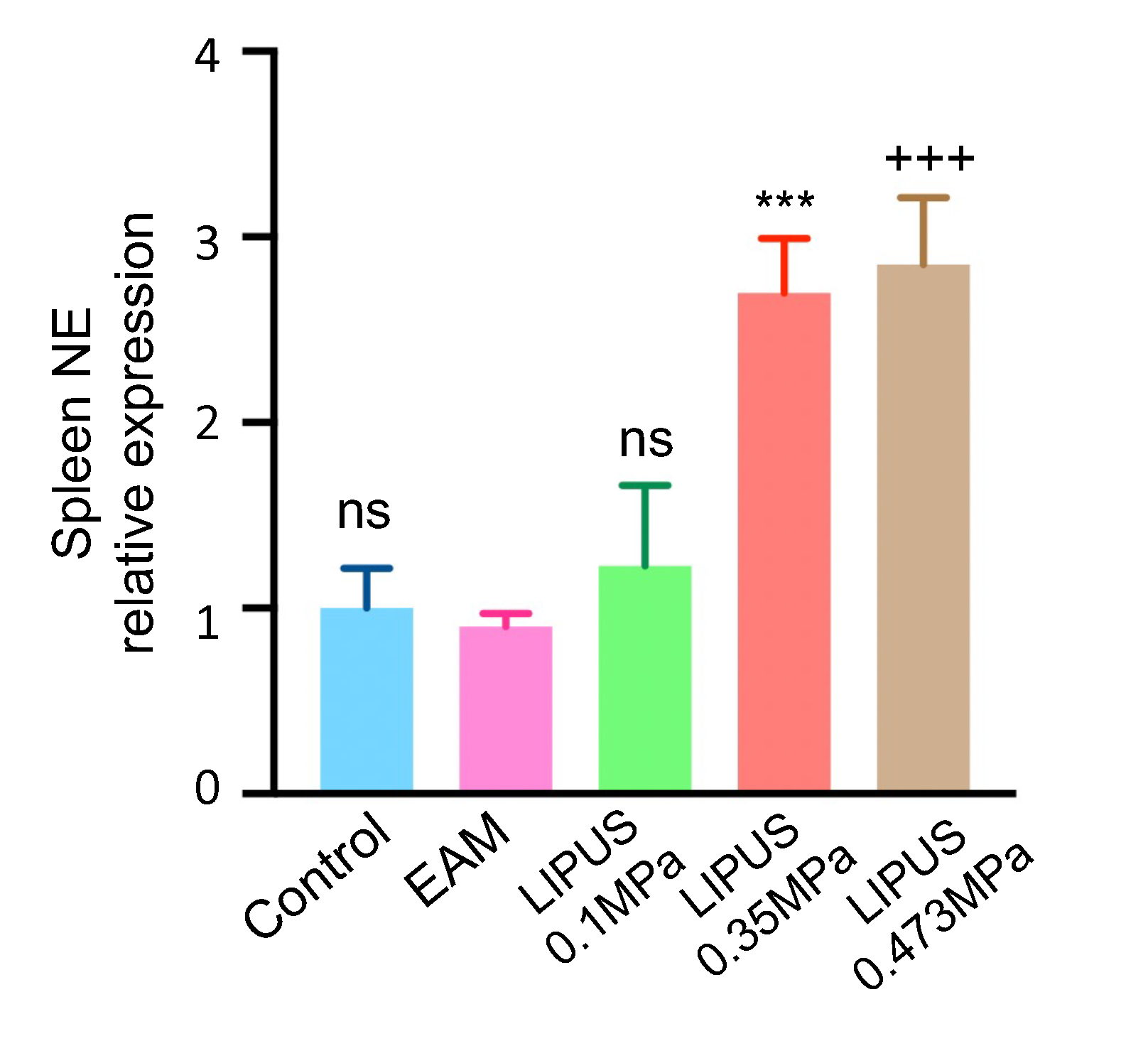

Supplement: Supplementary file 1 — Additional file 1. The expression of NE in spleen of EAM mice under stimulation of different acoustic pressure of LIPUS (n=5). ns represents control vs. EAM or LIPUS-0.1 MPa vs. EAM;* represents LIPUS-0.35 MPa vs. EAM; + represents LIPUS-0.473 MPa vs. EAM. (Data were shown as the mean ± SD, ordinary one-way analysis of variance, Tukey's multiple comparisons test; ###P or +++P < 0.001) EAM: Experimental Autoimmune Myocarditis; NE: norepinephrine; LIPUS: low-intensity pulsed ultrasound. [file 12974_2023_2773_MOESM1_ESM.tif]

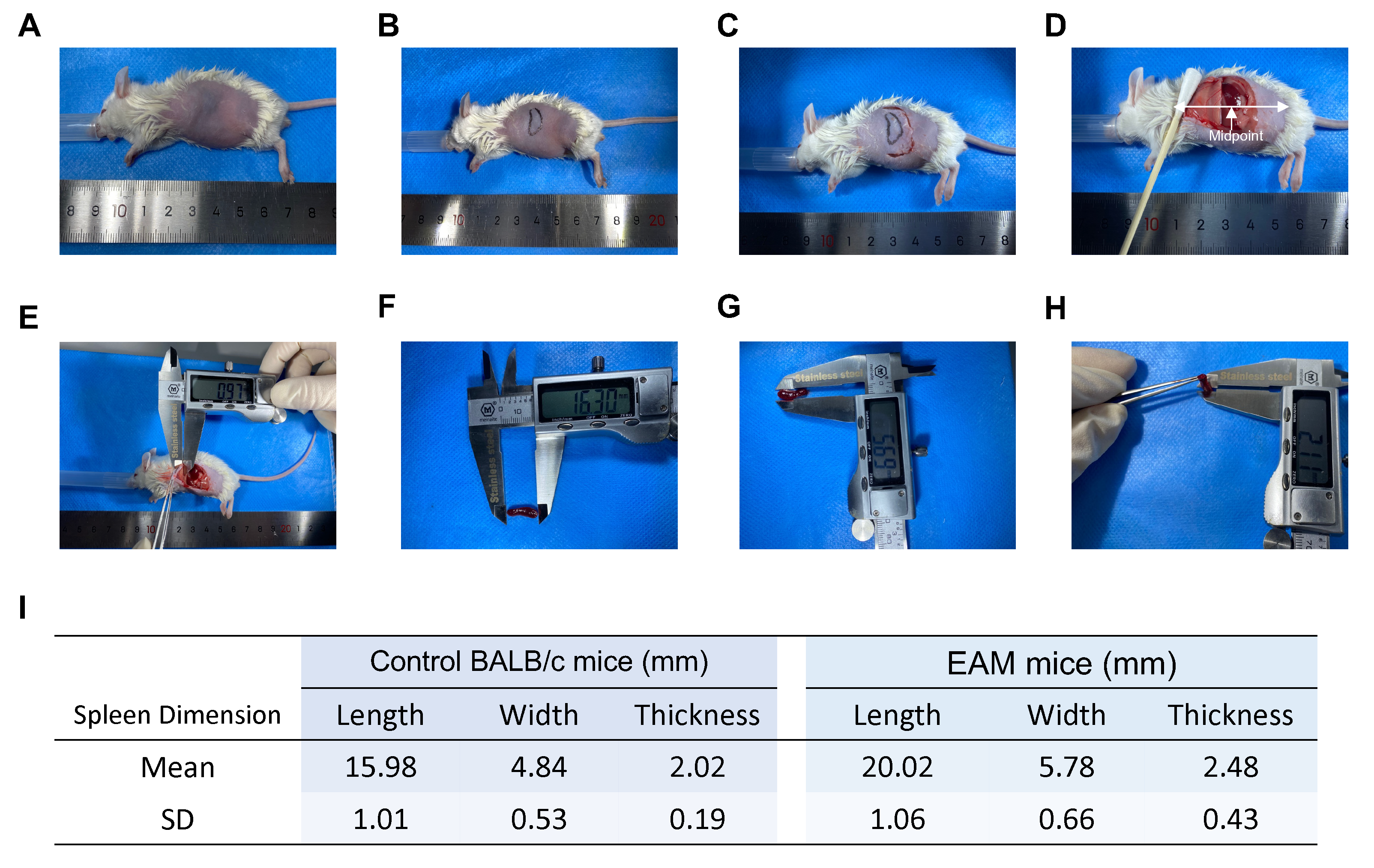

Supplement: Supplementary file 2 — Additional file 2. LIPUS stimulated localization on the spleen and measurement of mouse spleen dimensions. Mice were anesthetized with isoflurane before LIPUS stimulation. Since the spleen is shallow, it can be clearly outlined and marked through the thin skin (A-B). To confirm the spleen's location, the mouse skin was turned over (C). The spleen is consistently located halfway between the shoulder and hip joints of the animal, so the landmarks can be used to target the spleen (D). The depth (E), length (F), width (G) and thickness (H) of spleen were measured after euthanasia. These three-dimension data are recorded in mm in Table I (bottom). (n=5). LIPUS: low-intensity pulsed ultrasound. [file 12974_2023_2773_MOESM2_ESM.tif]

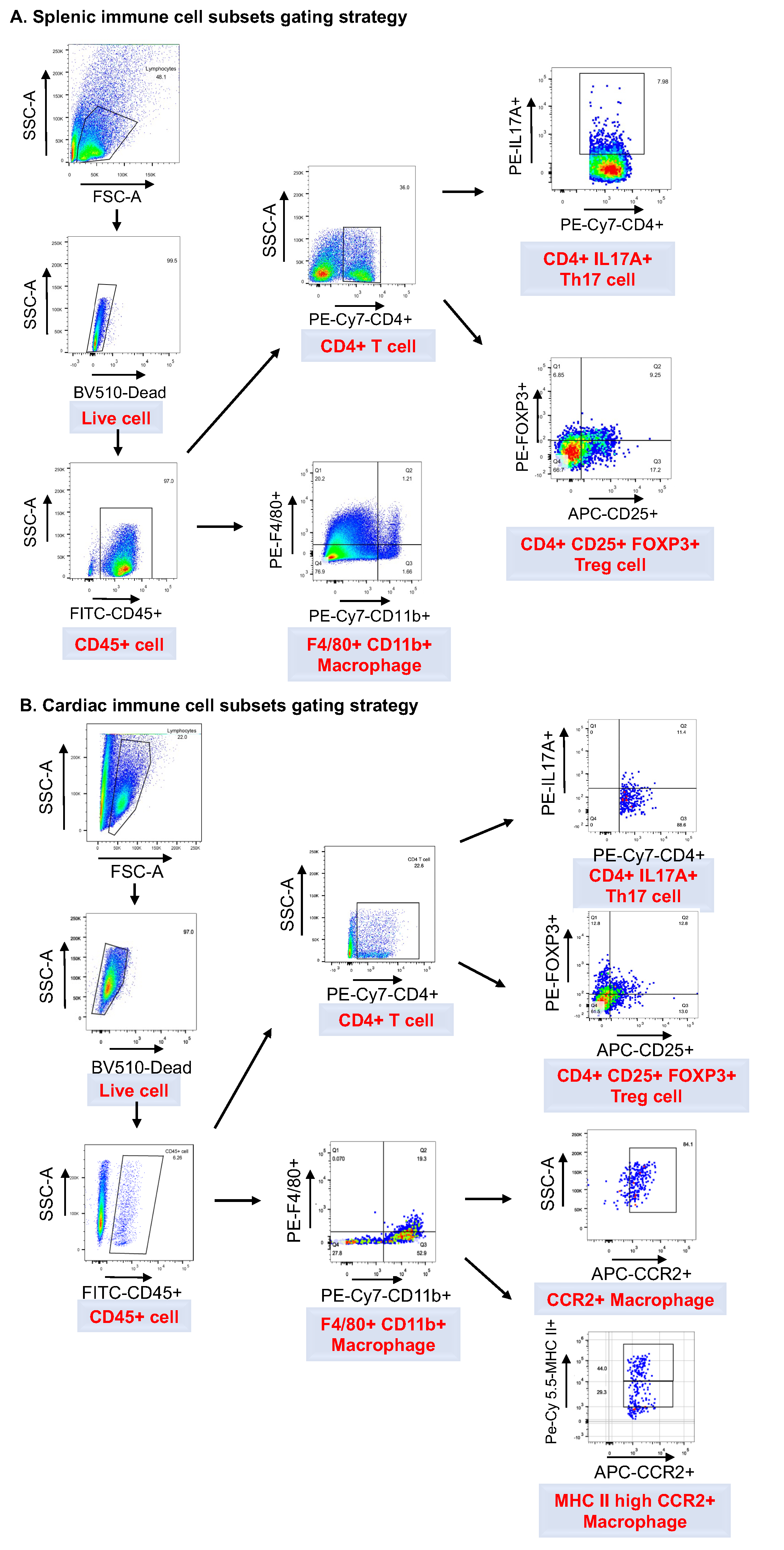

Supplement: Supplementary file 3 — Additional file 3. Cardiac and splenic immune cell subsets gating strategy. (A) Splenic T cell and macrophage gating strategy. (B) Cardiac T cell and macrophage gating strategy. FACS: Fluorescence Activated Cell Sorting. [file 12974_2023_2773_MOESM3_ESM.tif]

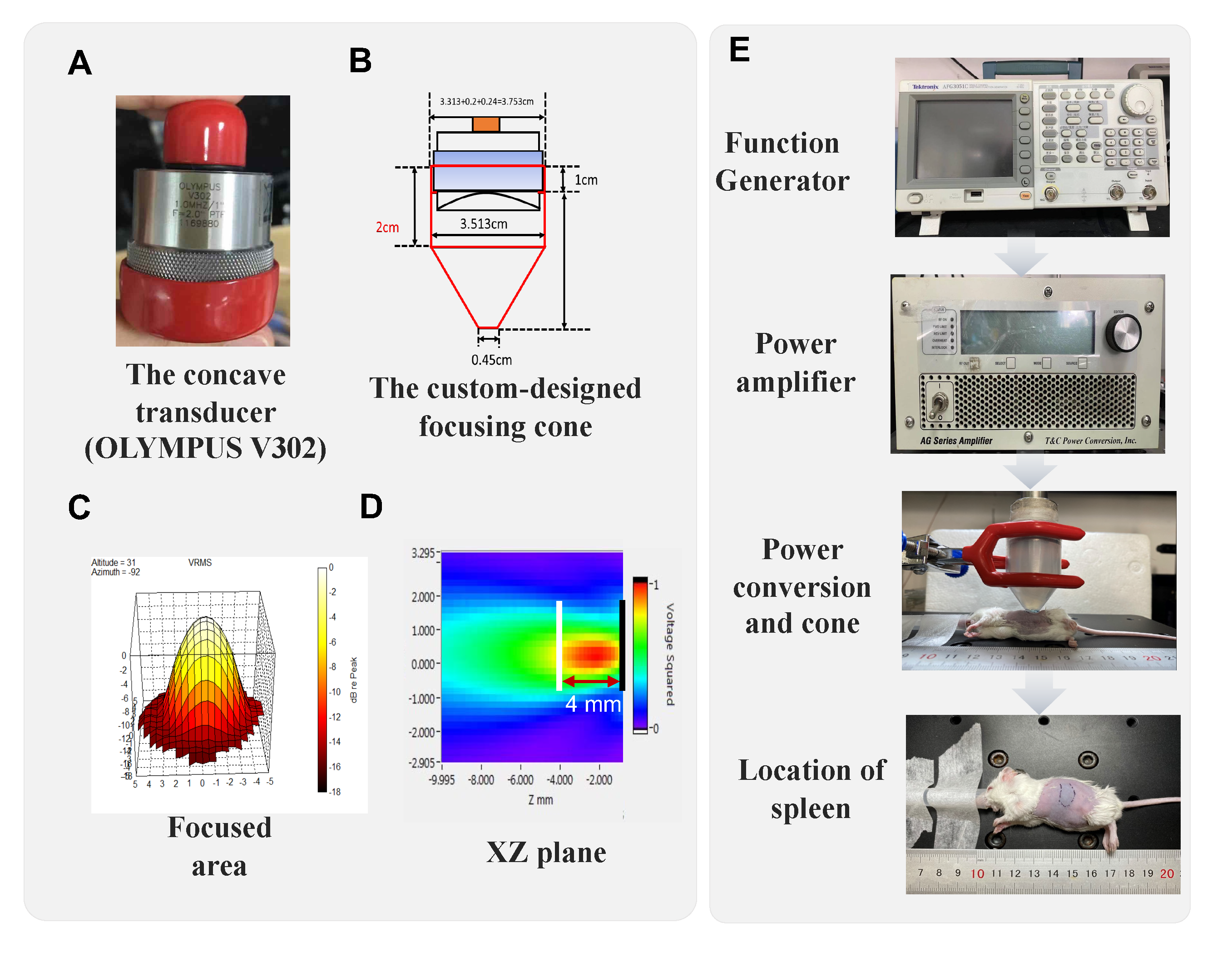

Supplement: Supplementary file 4 — Additional file 4. Sound field scanning of focused ultrasound transducer. (A) The concave transducer (OLYMPUS V302) with a custom-designed focusing cone (B) filled with agarose gel (1.5%, w/v). The ultrasound transducer’s sound field showed three-dimensional focused area (C) and focal depth (D). A black line shows the edge of the coupling cone; this is the level of contact with the skin. The focused sound field is distributed within 4mm depth (between black and white line). The system (E) consists of a Function Generator (Tektronix AFG3051C), a power amplifier (AG 1020, T&C Power Conversion, Inc.) and a concave transducer (OLYMPUS V302). [file 12974_2023_2773_MOESM4_ESM.tif]

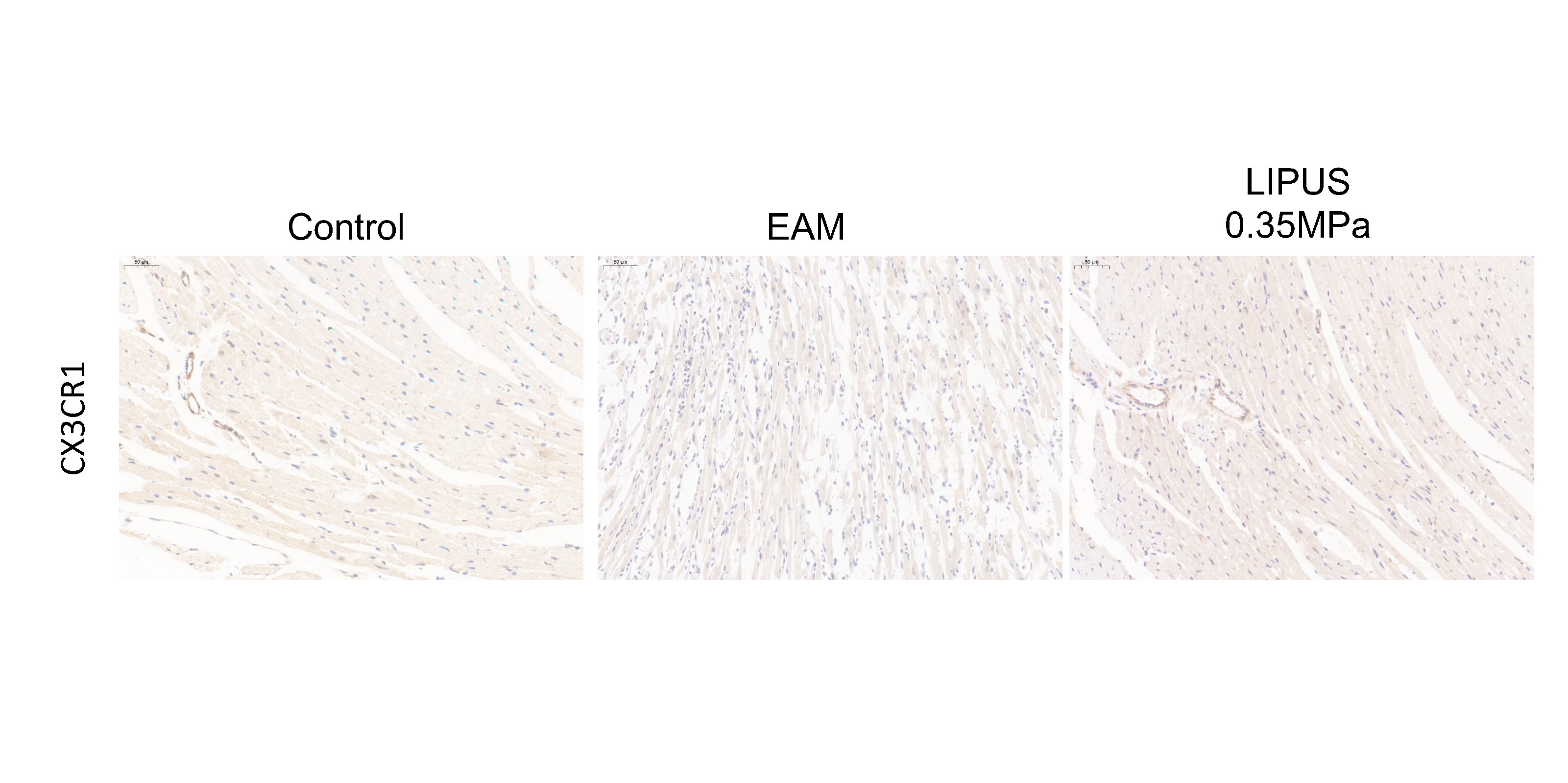

Supplement: Supplementary file 5 — Additional file 5. Effect of LIPUS on the immunohistochemical staining of CX3CR1 in myocardium. Representative immunohistochemical staining of CX3CR1 in myocardium of control, EAM, and LIPUS group (n=4). Scale bar represents 50 μm. EAM: Experimental Autoimmune Myocarditis; LIPUS: low-intensity pulsed ultrasound. [file 12974_2023_2773_MOESM5_ESM.tif]

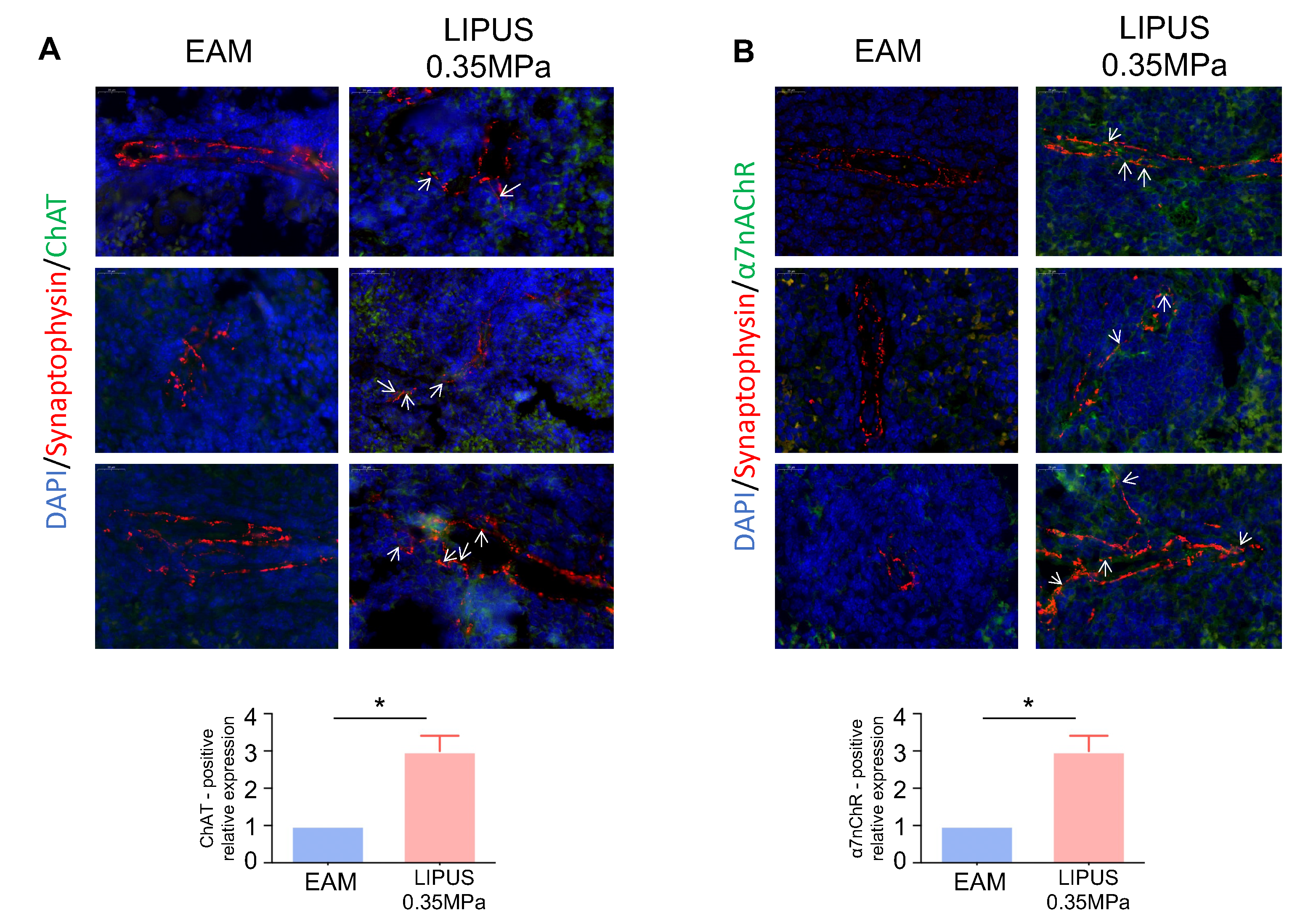

Supplement: Supplementary file 6 — Additional file 6. Nerve endings terminate adjacent to ChAT-positive or α7nAChR-positive cell in spleen. Synaptophysin-positive (green) nerve endings were found in close proximity to ChAT-positive (A: red) or α7nAChR-positive (B: red) in LIPUS group of mice (especially white arrow). In statistical analysis, the number of target cell in LIPUS group is normalized to EAM group (bottom). (n=4) * represents LIPUS-0.35 MPa vs. EAM. (Data were shown as the mean ± SD, unpaired T test, Mann-Whitney test; *P < 0.05) EAM: Experimental Autoimmune Myocarditis; LIPUS: low-intensity pulsed ultrasound; ChAT: choline acetyl transferase; α7nAChR: alpha7 nicotinic ACh receptor. [file 12974_2023_2773_MOESM6_ESM.tif]

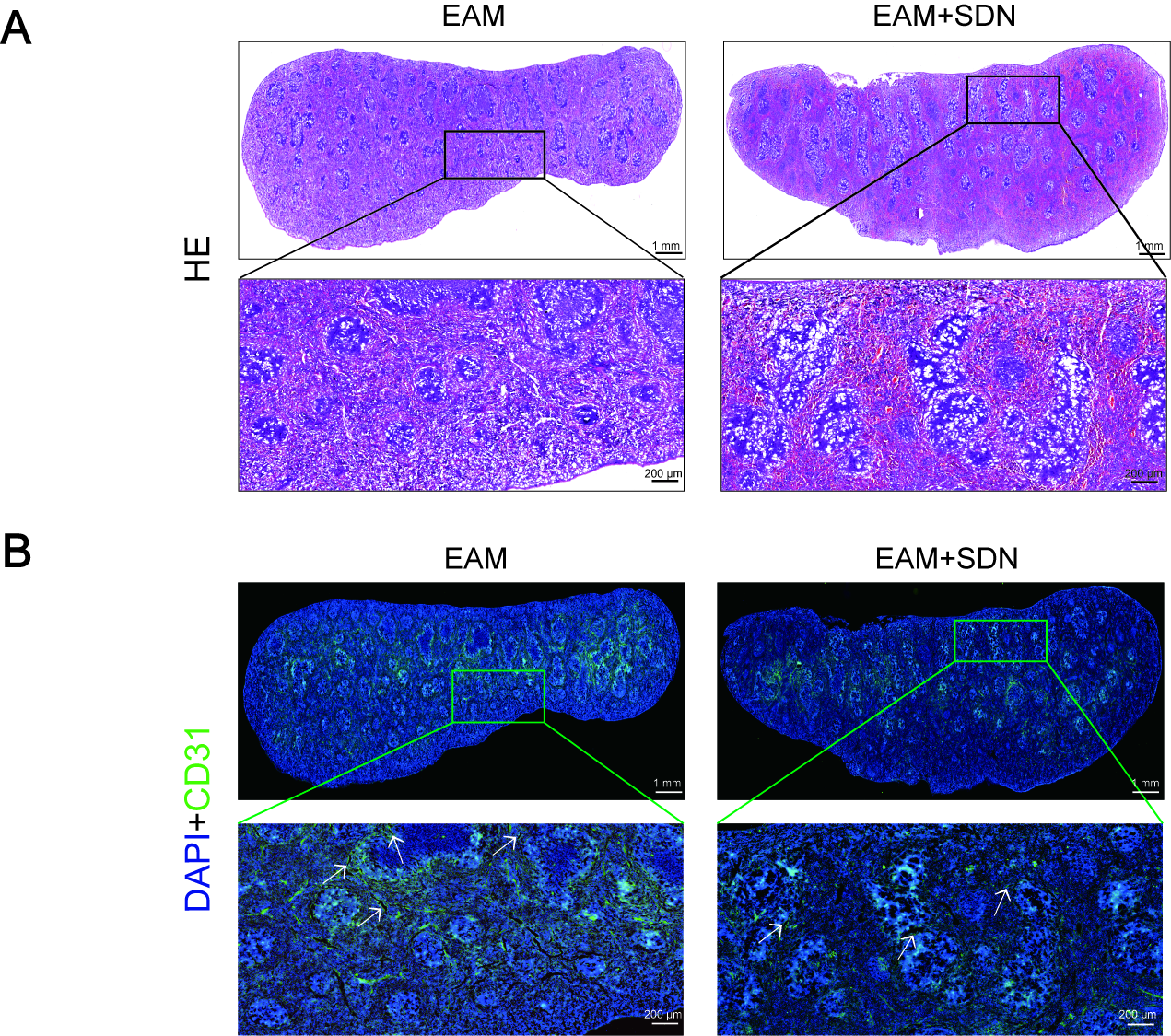

Supplement: Supplementary file 7 — Additional file 7. The effect of splenic denervation on vessels of EAM mice. A. HE staining for spleen histological signs of EAM mice and EAM+SDN mice. B. Representative images of whole spleen sections of EAM mice and EAM+SDN mice. Blue, nuclear staining. Green, CD31 is a sensitive and specific marker for vascular endothelial injury (especially white arrow). EAM: Experimental Autoimmune Myocarditis; SDN: Splenic Denervation; HE: hematoxylin and eosin. [file 12974_2023_2773_MOESM7_ESM.tif]
