# Supplementary material for: Noninvasive ultrasound stimulation to treat myocarditis through splenic neuro-immune regulation
Source: J Neuroinflammation. 2023 Apr 17;20:94. doi: 10.1186/s12974-023-02773-2 (PMC10108488; doi:10.1186/s12974-023-02773-2)
Supplement: Supplementary file 8 — Additional file 8. Table S1. Echocardiographic analysis of control, EAM and LIPUS treated mice groups on day 21. Summary of heart rate, ejection fraction, fractional shortening, left ventricular internal dimension diastolic (LVID diastolic), left ventricular internal dimension systolic (LVID systolic), left ventricular end-diastolic volume (LVEDV) and left ventricular end- systolic volume (LVESV) of health control mice, EAM mice and LIPUS mice after 21 days’ model, respectively. Data are presented as mean ± SD; ** P < 0.01; *** P < 0.001 versus Control; # P < 0.05; ## P < 0.01; ### P < 0.001 versus EAM; † P < 0.05; †† P < 0.01; ††† P < 0.001 versus Control. Table S2. Echocardiographic analysis of control, EAM and LIPUS treated mice groups on day 56. Summary of heart rate, ejection fraction, fractional shortening, left ventricular internal dimension diastolic (LVID diastolic), left ventricular internal dimension systolic (LVID systolic), left ventricular end-diastolic volume (LVEDV) and left ventricular end- systolic volume (LVESV) of health control mice, EAM mice and LIPUS mice after 56 days model, respectively. Data are presented as mean ± SD; *** P < 0.001 versus Control; ## P < 0.01; ###P < 0.001 versus EAM; †† P < 0.01; ††† P < 0.001 versus Control. Data are presented as mean ± SD. Table S3. Sequences of the primers for real time RT-PCR. Table S4 FACS Information of splenic and cardiac immune cells. [file 12974_2023_2773_MOESM8_ESM.docx]

**Table S1 Echocardiographic analysis of control, EAM and LIPUS treated mice groups on day 21:**

|  | **Control** | **EAM** | **LIPUS** |
| --- | --- | --- | --- |
| **Heart Rate (BPM)** | **401.90±16.60** | **284.60±53.50^**^** | **346.20±48.50^#^** |
| **Ejection Fraction (EF, %)** | **84.40±4.01** | **52.39±7.86^***^** | **70.37±4.94^###††^** |
| **Fractional Shortening (FS, %)** | **51.66±4.98** | **26.37±4.85^***^** | **38.42±3.76^##†††^** |
| **LVID diastolic (mm)** | **2.50±0.24** | **3.54±0.32^**^** | **2.89±0.52^#^** |
| **LVID systolic (mm)** | **1.23±0.13** | **2.58±0.28^***^** | **1.80±0.35^###††^** |
| **LVEDV (μL)** | **22.73±5.65** | **53.16±11.69^**^** | **33.52±13.50^#^** |
| **LVESV (μL)** | **3.65±1.03** | **24.69±6.12^***^** | **10.38±5.07^###†^** |

**Table S1 Echocardiographic analysis of control, EAM and LIPUS treated mice groups on day 21.** Summary of heart rate, ejection fraction, fractional shortening, left ventricular internal dimension diastolic (LVID diastolic), left ventricular internal dimension systolic (LVID systolic), left ventricular end-diastolic volume (LVEDV) and left ventricular end- systolic volume (LVESV) of health control mice, EAM mice and LIPUS mice after 21 days’ model, respectively. Data are presented as mean ± SD; ^**^ *P*﹤0.01; ^***^ *P*﹤0.001 versus Control; ^#^ *P*﹤0.05; ^##^ *P*﹤0.01; ^###^ *P*﹤0.001 versus EAM; ^†^ *P*﹤0.05; ^††^ *P*﹤0.01; ^†††^ *P*﹤0.001 versus Control.

**Table S2 Echocardiographic analysis of control, EAM and LIPUS treated mice groups on day 56.:**

|  | **Control** | **EAM** | **LIPUS** |
| --- | --- | --- | --- |
| **Heart Rate (BPM)** | **376.00±21.38** | **331.70±62.27** | **350.70±27.48** |
| **Ejection Fraction (EF, %)** | **70.62±3.48** | **42.45±5.20^***^** | **60.05±2.06^###†††^** |
| **Fractional Shortening (FS, %)** | **39.09±2.53** | **20.47±2.95^***^** | **31.20±1.35^###†††^** |
| **LVID diastolic (mm)** | **3.34±0.53** | **3.72±0.15** | **3.56±0.32** |
| **LVID systolic (mm)** | **2.03±0.39** | **2.95±0.15^***^** | **2.32±0.18^##††^** |
| **LVEDV (μL)** | **47.10±17.38** | **59.35±5.71** | **53.56±11.59** |
| **LVESV (μL)** | **14.08±6.29** | **33.7±4.17^***^** | **18.80±3.83^###^** |

**Table S2 Echocardiographic analysis of control, EAM and LIPUS treated mice groups on day 56.** Summary of heart rate, ejection fraction, fractional shortening, left ventricular internal dimension diastolic (LVID diastolic), left ventricular internal dimension systolic (LVID systolic), left ventricular end-diastolic volume (LVEDV) and left ventricular end- systolic volume (LVESV) of health control mice, EAM mice and LIPUS mice after 56 days model, respectively. Data are presented as mean ± SD; ^***^ *P*﹤0.001 versus Control; ^##^ *P*﹤0.01; ^###^ *P*﹤0.001 versus EAM; ^††^ *P*﹤0.01; ^†††^ *P*﹤0.001 versus Control. Data are presented as mean ± SD.

**Table S3 Sequences of the primers for real time RT-PCR:**

| Gens | primers | sequences |
| --- | --- | --- |
| IL-6 | Forward  Reverse | 5´- ACAACCACGGCCTTCCCTACT -3´  5´- CTCATTTCCACGATTTCCCAGA -3´ |
| RORγt | Forward  Reverse | 5´- GCAGGAGCAATGGAAGTCG -3´  5´- CGCTGAGGAAGTGGGAAAA -3´ |
| IL-17A | Forward  Reverse | 5´-GCTGTTGCTGCTGCTGAG-3´  5´- TGGAACGGTTGAGGTAGTC -3´ |
| IL-21 | Forward  Reverse | 5´- GGACCCTTGTCTGTCTGGTAG-3´  5´- TGTGGAGCTGATAGAAGTTCAGG-3´ |
| IL-22 | Forward  Reverse | 5´- ATGAGTTTTCCCTTATGGGGAC-3´  5´- GCTGGAAGTTGGACACCTCAA-3´ |
| IL-23 | Forward  Reverse | 5´- ATGCTGGATTGCAGAGCAGTA-3´  5´- ACGGGGCACATTATTTTTAGTCT-3´ |
| Foxp3 | Forward  Reverse | 5´- GAGAAAGCGGATACCAAA-3´  5´- TGTGAGGACTACCGAGCC-3´ |
| TGF-β | Forward  Reverse | 5´- CAAACTAAGGCTCGCCAGTCC -3´  5´- TTGCGGTCCACCATTAGCAC -3´ |
| IL-10 | Forward  Reverse | 5´- GGACAACATACTGCTAACCGACTC -3´  5´- TTCATGGCCTTGTAGACACCTT -3´ |
| IL-35 | Forward  Reverse | 5´- TATGGTCAGCGTTCCAACAGC-3´  5´- TTCGGGACTGGCTAAGACACC-3´ |
| CCL-3 | Forward  Reverse | 5´- CATATGGAGCTGACACCCCG-3´  5´- GAGCAAAGGCTGCTGGTTTC-3´ |
| CCL-4 | Forward  Reverse | 5´- TGTGCAAACCTAACCCCGAG-3´  5´- CCATTGGTGCTGAGAACCCT-3´ |
| CCL-5 | Forward  Reverse | 5´-GTGCTCCAATCTTGCAGTCG -3´  5´- GGATTACTGAGTGGCATCCCC-3´ |
| IL-12 | Forward  Reverse | 5´- TGGTTTGCCATCGTTTTGCTG-3´  5´- ACAGGTGAGGTTCACTGTTTCT-3´ |
| IL-15 | Forward  Reverse | 5´-GGAGCTGGAGGAGAAAACCTT -3´  5´- AACACGGAAGAGGCTCGCAT-3´ |
| IL-18 | Forward  Reverse | 5´- TCTTGGCCCAGGAACAATGG -3´  5´- ACAGTGAAGTCGGCCAAAGT-3´ |
| CXCL-10 | Forward  Reverse | 5´- AAGCTATGTGGAGGTGCGAC-3´  5´- AACCCCTTGGGAAGATGGTG-3´ |
| IL-1β | Forward  Reverse | 5´- AAATACCTGTGGCCTTGGGC-3´  5´- CTTGGGATCCACACTCTCCAG-3´ |
| TNF-α | Forward  Reverse | 5´- CATCTTCTCAAAATTCGAGTGACAA-3´  5´- TGGGAGTAGACAAGGTACAACCC-3´ |
| IFN-γ | Forward  Reverse | 5´-GCAACAGCAAGGCGAAAAAG -3´  5´- CGCTTCCTGAGGCTGGATTC-3´ |
| BNP | Forward  Reverse | 5´- CTGAAGGTGCTGTCCCAGAT -3´  5´- CCTTGGTCCTTCAAGAGCTG -3´ |
| MMP2 | Forward  Reverse | 5′-ACACCAAGAACTTCCGACTATCCAATG-3′  5′-CAGTACCAGTGTCAGTATCAGCATCAG-3′ |
| MMP9 | Forward  Reverse | 5′-CTCCTGGTGCTCCTGGCTCTAG-3′  5′-GTGTAACCATAGCGGTACAGGTAATCC-3′ |
| AChE | Forward  Reverse | 5´- ATCGGTGTACCCCAAGCAAG-3´  5´- CTCGTCCAGAGTATCGGTGG-3´ |
| ChAT | Forward  Reverse | 5'-TTCTGCTGTTATGGCCCTGTGGTA-3'  5´- ACTTGGGCTGTCTTTGTGCATGTG-3' |
| CHT1 | Forward  Reverse | 5´- GAAAACCAAAAACAGCGGCAA-3´  5´- AGGACCCTCTGGAAGTAGGC-3´ |
| VAChT | Forward  Reverse | 5´- GTGCGTTGCACTGTTACTGG-3´  5´- GACTGTGGAGGCGAACATGA-3´ |
| β-actin | Forward  Reverse | 5´- GTGACGTTGACATCCGTAAAGA-3´  5´- GTAACAGTCCGCCTAGAACAC-3´ |

**Table S4 FACS Information of splenic and cardiac immune cells**

| **FACS Information of splenic immune cells** | | |
| --- | --- | --- |
| Fluorochromes | FACS Antigens | ID |
| BV-510 | Zombie Aqua™ Fixable Viability Kit | Biolegend: 423101 |
| FITC | anti-mouse CD45 | Biolegend: 103108 |
| PE/Cyanine7 | Rat anti-mouse CD4 | BD PMG: 561099 |
| PE/Cyanine7 | anti-mouse/human CD11b | Biolegend: 101215 |
| PE | anti-mouse F4/80 | Biolegend: 123109 |
| PE | Ms IL17A TC11-18H10 | BD PMG: 561020 |
| PE | Anti-mouse/rat FOXP3 (FJK-16S) | eBioscience: 12-5773-80 |
| APC | MS CD25 MAB | BD PMG: 557192 |
| **FACS Information of cardiac immune cells** | | |
| Fluorochromes | FACS Antigens | ID |
| BV-510 | Zombie Aqua™ Fixable Viability Kit | Biolegend: 423101 |
| FITC | Anti-mouse CD45 | Biolegend: 103108 |
| PE/Cyanine7 | Rat anti-mouse CD4 | BD PMG: 561099 |
| PE/Cyanine7 | Anti-mouse/human CD11b | Biolegend: 101215 |
| PE | Anti-mouse F4/80 | Biolegend: 123109 |
| PE | Ms IL17A TC11-18H10 | BD PMG: 561020 |
| PE | Anti-mouse/rat FOXP3 (FJK-16S) | eBioscience: 12-5773-80 |
| APC | MS CD25 MAB | BD PMG: 557192 |
| Alexa Fluor® 647 | Anti-mouse CD192 (CCR2) | Biolegend:150603 |
| PerCP/Cyanine5.5 | Anti-mouse I-A/I-E (MHC II) | Biolegend: 107625 |
